# Pyrolysis Compound-Specific Isotope Analysis of Proteins in Aged Paintings: Diketopiperazine δ^13^C Values as Collagen Source Indicators

**DOI:** 10.1021/acs.analchem.5c07901

**Published:** 2026-07-15

**Authors:** Eugenia Geddes da Filicaia, David A. Peggie, Iain P. Kendall, Ian D. Bull, Richard P. Evershed

**Affiliations:** † Organic Geochemistry Unit, School of Chemistry, 1980 University of Bristol, Bristol BS8 1TS, U.K.; ‡ Scientific department, The National Gallery, Trafalgar Square, London WC2N 5DN, U.K.

## Abstract

Proteins have been used as adhesives in art throughout history, but determining their source in aged paintings is challenging. Moreover, the distinction between terrestrial and aquatic animal glues is only achievable with proteomic approaches. Compound-specific isotope analysis (CSIA) of collagen, used in the neighboring field of archeology for analogous purposes, has so far required too much sample to be used for painted objects. However, this paper presents a methodology that enables a reduction of sample size by about 3 orders of magnitude (from mg to μg), widening the application of CSIA to rare and fragile objects in our cultural patrimony. Based on direct inlet pyrolysis-gas chromatography-combustion-isotope ratio mass spectrometry (DIP-GC-C-IRMS), the method targets the δ^13^C values of pyrolytic protein markers, especially diketopiperazines (DKPs), reported here for the first time. The isotopic imprint of modern references for rabbit skin and isinglass glues, casein, and chicken and duck eggs was confirmed through established elemental analysis (EA)-IRMS and GC-C-IRMS methods. As expected, bulk and individual amino acid (AA) δ^13^C and δ^15^N values for terrestrial animal glues exhibited a higher depletion than those for marine glues, a relationship retained by the DKP δ^13^C values. Furthermore, the ground layers of two 16th-century Italian paintings from the National Gallery collection yielded δ^13^C_DKP_ values consistent with terrestrial animal glue, confirming that the isotopic signature of these compounds is retained during aging and degradation.

## Graphical abstract

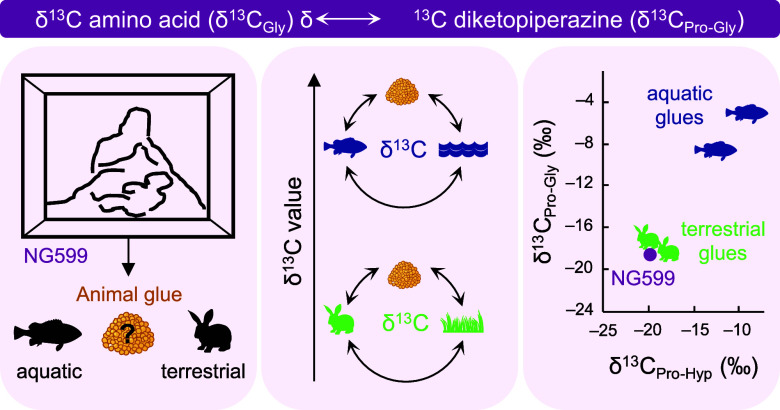

Proteins are an important class of organic compounds frequently encountered in cultural heritage objects. Due to their excellent film-forming and adhesive properties, these materials have been used since ancient times.^1,2^ In paintings, milk (casein), egg, and animal glue proteins are commonly identified, with the earliest known examples dating to the Upper Paleolithic (40,000–10,000 BCE), in cave paintings in Africa, Asia, and Europe.^3^ Whole egg, or just the yolk, has been most typically used as a paint binder, while egg albumin, or glair, has functioned as a glue for gilding, a primer for plaster, a varnish, and as a medium for retouching.^4–6^ Similarly, casein is still often employed in conservation practices,^6^ whereas it was historically widely used in murals,^1^ particularly for painting with blue pigments.^5^ Animal glue, also called size, was employed in ground layers in easel paintings,^1^ to prepare the surface in wall paintings, as a paint binder, a mordant for gilding,^5^ and a consolidant.^7^ The Roman scholar Plinius (1st century CE) describes two types of animal glues: *taurokolla*, made from bull skin, and *ichtyokolla*, made from fish. However, medieval painting treatises discuss the use of glue made from various animals, including ox or cow, rabbit, and fish.^5^ In an 8th-century CE manuscript from the Cathedral of Lucca, the latter is specifically indicated to illuminate manuscripts^8^ and may have been used more widely than currently known due to its particular characteristics. Isinglass, a glue prepared from the swim bladders of (traditionally) sturgeon,^9^ forms a clearer film upon drying than terrestrial animal glues^7^ and remains more elastic, flexible, and tough.^7,10^

Because of their complex macromolecular and highly degraded nature, the analysis of proteins in cultural heritage presents considerable challenges. With aging and exposure to environmental agents, proteins undergo physical and chemical changes that are still not fully understood.^6,11^ In addition, owing to ethical constraints, samples (if available) are scarce and minute, and surviving proteins are found in low concentration within a heterogeneous inorganic matrix. Proteomics, which can identify peptide fragments, is a powerful technique for protein identification in this field. However, these approaches are not widely accessible and are particularly prone to contamination. Analysis and data interpretation are time-consuming, and results can be ambiguous, as the available databases do not necessarily contain ancient species.^12^

More typically, proteins in paintings are considered in terms of their amino acid (AA) composition, observable by gas chromatography-mass spectrometry (GC-MS) after hydrolysis, and protein identification is achieved through quantitative analysis.^1^ However, in practice, this is often unattainable, as multiple sources of AAs might be present within the paint sample,^13^ both as original and added (conservation) materials. Hydroxyproline (Hyp), present (almost) exclusively in collagen, enables the unambiguous identification of animal glue, but determination of animal species and identification of fish glues, in particular,^12^ remain difficult. These have been reported to exhibit a lower proportion of proline (Pro) and Hyp compared to terrestrial animal glues,^14^ which have been tentatively used to identify fish glue in the ground layer of Tanaquil, painted around 1519 by Beccafumi.^15^

Analytical pyrolysis (Py), presenting many advantages widely discussed in the literature,^16,17^ has become a popular alternative technique for the study of these materials. As proteins tend to generate intricate pyrograms,^18^ differentiation between protein binders relies on detection of specific markers, such as cyclic dipeptides.^19^ In particular, 2,5-diketopiperazines (DKPs), formed through thermal cyclization of neighboring AAs in the peptide chain, have been exploited as such identifiers in complex matrices in art, archeology,^20,21^ forensic science, and biology.^22^ Even though the likelihood of formation of DKPs from aged paint samples is believed to decrease with age,^21^ DKPs were obtained from the analysis of bog bodies from the Iron Age,^23^ and from archeological tissues (40,000–50,000 years old) found in an Egyptian tomb, the desert, and the Siberian permafrost.^24,25^ Recently, these compounds were also obtained from Py-GC×GC-time-of-flight (TOF)-MS of fossilized fish scales from the Pliocene and Mesozoic.^26^

The complexities and challenges presented by the analysis of protein paint binders illustrate the need for novel methods of identification for these materials, in particular to distinguish between terrestrial and fish-animal glues. An alternative approach with great potential may be found in the neighboring field of archeology, where collagen in bones and teeth has been used as a palaeodietary indicator since the 1970s. Determination of bulk δ^15^N values may suggest the position of an organism in the respective food web, while δ^13^C values offer insight into their diet.^27–29^ Bulk δ^13^C and δ^15^N values of collagen are weighted averages of the δ^13^C and δ^15^N values of their constituent AAs, which reflect both dietary sources and metabolic processes. Serine, glycine, and alanine (Ser, Gly, and Ala) are part of the glucogenic AA group, which is biosynthesized from intermediates of glycolysis. As pyruvate is the end product of glycolysis, considered the catabolic pathway of carbohydrates, the isotopic signature of these AAs should reflect that of dietary carbohydrates. Aspartic acid and glutamic acid (Asp and Glu), asparagine (Asn), and Pro are part of the ketogenic AA group, originating from intermediates of the tricarboxylic acid (TCA) cycle. Due to their TCA cycle precursors, the isotopic signature of these AAs should reflect that of the whole diet, if biosynthesized *de novo*. Finally, Hyp and hydroxylysine cannot be incorporated into proteins from the diet and are formed after the polypeptides are synthesized, during posttranslational processing of collagen. Hydroxylysine is formed from lysine (Lys), whereas Hyp results from hydroxylation of Pro residues.^30^ Moreover, AAs in the “trophic group“ tend to display a large enrichment in ^15^N with increasing trophic level, unlike those belonging to the “source group.“

Significant limitations therefore surround the interpretation of bulk stable isotope ratios, which, however, may be overcome by compound-specific isotope analysis (CSIA). Individual AA δ^13^C values have proven invaluable for distinguishing terrestrial from marine diets of our ancestors,^30–35^ while AA δ^15^N values are being increasingly used in archeological trophic position studies.^36–39^ Despite the similarity in target material, CSIA has never been extended to the analysis of animal glue, or any other protein, in paintings. Reasons for this are likely linked to the sample sizes required for standard protocols for GC-combustion-isotope ratio MS (GC-C-IRMS), which, however, as illustrated by recent studies,^40–42^ may be significantly reduced by undertaking direct inlet pyrolysis (DIP)-GC-C-IRMS. Here, this technique is applied to the analysis of proteins of paintings, in particular by targeting, for the first time, the stable carbon isotope composition of DKPs, protein pyrolysates. Having confirmed that DKPs reflect the isotopic signature of their constituent AAs, DKP δ^13^C values may be used to distinguish terrestrial from aquatic animal glue in aged paintings.

## Materials and Methods

### Standards and Samples

An AA mixture was prepared comprising 14 AA standards (each 1 mg mL^–1^ in 0.1 M HCl) of known δ^13^C and δ^15^N values, containing Ala, arginine (Arg), Asp, Glu, Gly, Hyp, leucine (Leu), Lys, phenylalanine (Phe), Pro, Ser, threonine (Thr), tyrosine (Tyr), and valine (Val). A separate solution containing norleucine (Nle) (Sigma-Aldrich, UK) was used as an internal standard (IS). Standard solutions (0.1 μg mL^–1^ in H_2_O) containing a single AA each were also prepared for Gly, isoleucine (Ile), Hyp, Pro, and Val. Gelatin (G1), casein (C1), egg white (EW1), rabbit skin glues (GRS1 and GRS2), and isinglass glues (GI1 and GI2) were obtained from the National Gallery reference material collection. Modern references of chicken and duck egg white (EWC and EWD, respectively) and egg yolk (EYC and EYD, respectively) spreads were made from eggs acquired from organic farms, and a casein reference standard (C2) was obtained from the Organic Geochemistry Unit archives. Samples of the ground layer from Madonna of the Meadow (NG599, Giovanni Bellini, ca. 1500–5) and from Tanaquil (NG6368, Domenico Beccafumi, ca. 1519) originate from the National Gallery sample archive.

### AA Derivatization for GC-C-IRMS Analysis

Protein references EWC, EWD, C2, GI1, GI2, GRS1, and GRS2 (3 mg each) were hydrolyzed into their constituent AAs (4 mL 6 M HCl_(aq)_, 100 °C, 24 h), which were derivatized into their *N*-acetyl isopropyl esters according to published protocols.^39,43,44^ AAs were converted to their isopropyl esters through the addition of a mixture of isopropanol and acetyl chloride (4:1 *v/v*, 0.5 mL, 100 °C, 1 h). The solutions were blown to dryness under a gentle stream of N_2_ (40 °C). Dichloromethane (DCM) was added (0.25 mL × 2) to remove residual reagents under N_2_. A mixture of acetone, triethylamine, and acetic anhydride (5:2:1 *v/v/v*), 1 mL, 60 °C, 10 min) was then added to the AA isopropyl esters. Reagents were removed under a gentle stream of N_2_ at room temperature, and saturated NaCl_(aq)_ (1 mL) was added. The derivatized AAs were extracted into ethyl acetate (2 mL × 3), which was evaporated under a very gentle flow of N_2_ at room temperature. Residual water was removed by the addition of DCM (1 mL × 3) evaporated under a gentle stream of N_2_ in an ice bath. The AA *N*-acetyl isopropyl ester derivatives were redissolved in ethyl acetate for screening by GC-flame ionization detection (GC-FID) and then analyzed by GC-C-IRMS.

### DIP-GC-MS and DIP-GC-C-IRMS of Proteinaceous Samples and AA Standards

Reference proteinaceous binders and paint samples (a few μg, down to 1 or less for pure undegraded protein) were placed in a furnaced (450 °C, 1 min) thermal separation probe (TSP) tube, and analyzed by DIP-GC-MS or DIP-GC-C-IRMS. When derivatization was undertaken (DIP-GC-MS only), tetramethylammonium hydroxide (TMAH) (1.5 μL, 2.5% in methanol) was added to the TSP tube, and the solvent was allowed to evaporate at room temperature before analysis. AA single standard solutions, either two different AAs (1 μL each) or one AA (2 μL), were loaded by a microsyringe into TSP tubes. The tubes were left in a desiccator overnight to remove the solvent prior to analysis.

### Instrumental Analyses

For elemental analysis (EA)-IRMS, GC-FID, and GC-C-IRMS methods, please see SI1. All chromatograms were replotted in RStudio. DIP-GC-MS was carried out on an Agilent Technologies 7890A GC coupled to an Agilent Technologies 5975C MSD (Agilent Technologies, Santa Clara, CA, USA), a single quadrupole MS. The GC was equipped with a nonpolar fused-silica capillary column (30 m × 0.25 mm i.d. × 0.25 μm film thickness) with a BPX5 stationary phase (5% phenyl polysilphenylene-siloxane, SGE) and a TSP in the multimode inlet (MMI), operated in splitless mode. The initial TSP temperature was 50 °C, set to rise to 450 °C (900 °C min^–1^, 3 min isothermal hold), before cooling to 250 °C (25 °C min^–1^, 40 min isothermal hold). The oven temperature began at 40 °C, was increased to 200 °C (10 °C min^–1^), and then to 310 °C (6 °C min^–1^) with a final hold time of 20 min. He was used as a carrier gas at a constant flow (1.2 mL min^–1^).

The MS was set to acquire in full scan mode (*m*/*z* 50–650) at 0.2 scan s^–1^, and was operated at an EI potential of 70 eV with a solvent delay of 6.5 min. The MS transfer line temperature was set to 280 °C, the ion source temperature to 230 °C, and the quadrupole to 150 °C. Data acquisition was undertaken using Agilent Technologies Chemstation, and data processing with Agilent Technologies MassHunter software (version 10.0) and the AMDIS deconvolution software (NIST, version 2.66). Peaks were identified using a combination of mass spectral information, GC retention times (RTs), and comparison to the NIST mass spectral library (Mass Spectral Search Program version 2.0 and NIST 08 MS Library), published data sets, and NG databases. An *n*-alkane “ladder“ (with a characteristic bell-shaped peak profile) standard, containing C_7_–C_40_ saturated alkanes (0.01 mg mL^–1^ in *n*-hexane), was used to monitor instrument performance.

DIP-GC-QTOF-MS analysis was carried out on an Agilent Technologies 7890B GC coupled to an Agilent Technologies 7200 Accurate Mass QTOF MS (Agilent Technologies, Santa Clara, CA, USA). The same method was used, except that the MMI was held at 450 °C for 1 min only. The MS was set with an acquisition rate of 5 spectra s^–1^, an acquisition time of 200 ms spectrum^–1^, and an emission current of 2.1 μA. DIP-GC-C-IRMS analysis was carried out on an Agilent Technologies 8890 GC coupled to an Elementar isoprime precisION IRMS via an Elementar GC5 combustion interface, using a combustion reactor containing CuO pellets and Ag wool (Elementar Analysensysteme GmbH, Hanau, Germany), maintained at 850 °C. The GC parameters were identical to those for DIP-GC-MS, except that the MMI, after being held at 450 °C for 3 min, was cooled to 300 °C (900 °C min^–1^, isothermal hold for 50 min). Additionally, the oven program was typically shortened by setting a lower maximum temperature (280 °C instead of 310 °C), where it was held for 10 min. He was used as a carrier gas at a constant flow (2 mL min^–1^).

The MS was equipped with three Faraday cups collecting for the masses *m*/*z* 44 (^12^C^16^O_2_), 45 (^13^C^16^O_2_ and ^12^C^17^O^16^O), and 46 (^12^C^18^O^16^O). The solvent delay was 8 min, and data acquisition and processing were undertaken using the lyticOS software (Elementar, version 5.0.4.118). All δ^13^C values are reported relative to reference CO_2_ of a known isotopic composition, introduced directly in the ion source in two pulses at the start and two pulses at the end of each analysis. An in-house quality-control standard containing *n*-alkanes (C_24:0_, C_28:0_, C_30:0_, and C_32:0_) with known δ^13^C values, analyzed under identical conditions as the investigated compounds, was used to calibrate the instrument through a two-point normalization. The reference standard was also used to monitor instrument performance. All sample δ^13^C values are the mean of duplicate analyses.^41^

## Results and Discussion

### Pyrolytic Marker Identification from DIP-GC-MS of Protein References

The thermal degradation of intact proteins has been shown to be a multistep reaction,^45^ where the formation of DKPs, occurring in the first degradation step (*T*_max_ = 325 °C), does not require high Py temperatures.^21^ Protein markers may therefore be detected through DIP-GC-MS, as testified by the analysis of a soapstone fragment from Qarmaaluit (18th–19th century CE).^46^ Nonetheless, given that this technique is not widely reported in the literature, modern protein references were analyzed by DIP-GC-MS, and pyrolytic profiles obtained were compared to published Py-GC-MS data. Studies have been undertaken with^22,47,48^ and without^22,26^ TMAH, hence, both methods were carried out here. Results were scrutinized, in particular, to identify pyrolytic markers suitable for DIP-GC-C-IRMS analysis. Egg yolk, which presents a similar AA composition as egg white, was not examined, as its high fat content was deemed to interfere with the analysis.

In general, DIP-GC-MS analyses involving derivatization with TMAH, in particular for animal glues, featured many more compounds compared to the total ion chromatograms (TICs) obtained without derivatization (see Figure S1 for G1, aged gelatin spread). In this case, GC-MS TICs exhibited better baseline separation with the most abundant peaks corresponding to the targeted DKPs. Further analysis of glue samples was therefore carried out without derivatization, as good baseline resolution is central to reliable compound-specific stable isotope determinations.^43^ Analysis of rabbit skin glues (GRS1 and GRS2) and isinglass glues (GI1 and GI2) yielded GC-MS TICs identical to each other. In accordance with the literature,^21,22,49^ as illustrated in Figure 1a, they are dominated by peaks corresponding to diketodipyrrole (peak 8) and cyclo­(Pro-Gly) (peak 10). Because of its relatively high polarity, or overloading, the latter produces a broad (fronting) peak.^50^

**Figure 1:**
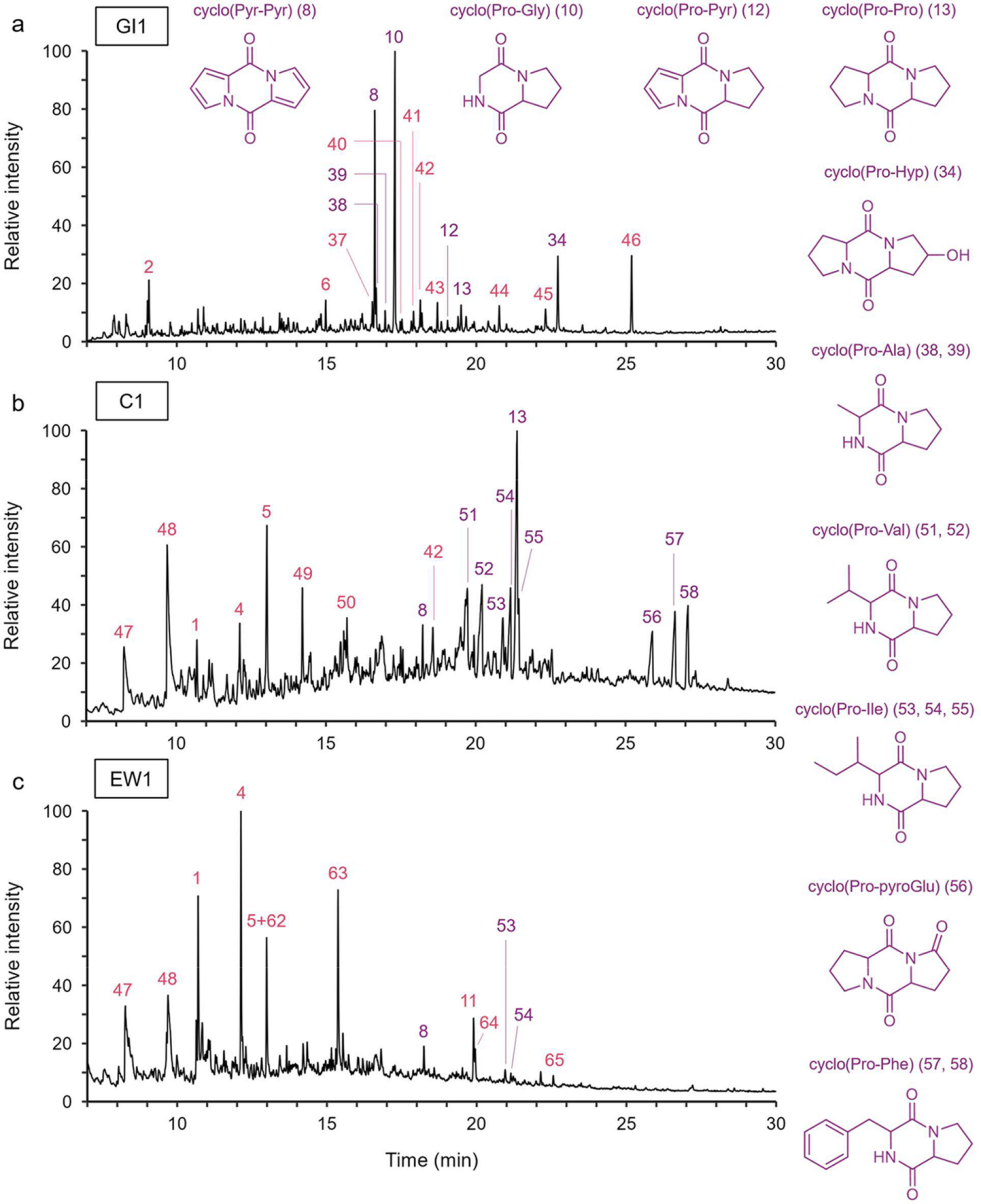
Partial DIP-GC-QTOF-MS total ion chromatograms (TICs) for (a) isinglass, GI2, (b) casein, C1, and (c) egg white, EW1. Information on relevant DKPs (in purple) and other peaks (in pink) is listed in Table S1.

Between these, cyclo­(Pro-Ala) is present as two isomers (peaks 38 and 39). In the DIP-GC-MS TIC, peak 38 is coeluting with an unknown compound (37), while this peak is better separated in DIP-GC-QTOF-MS. These are followed by cyclo­(Pro-Pyr) (peak 12) and cyclo­(Pro-Pro) (peak 13). With the exception of Glu, not prone to DKP formation, the cyclic dipeptides observed parallel the AAs present in the highest amounts in collagen. In addition, they are often adjacent in the collagen peptidic chains,^51^ favoring this kind of reaction. A proposed reaction scheme for the formation of DKP from the pyrolysis of collagen is shown in Scheme 1.

**Scheme 1:**
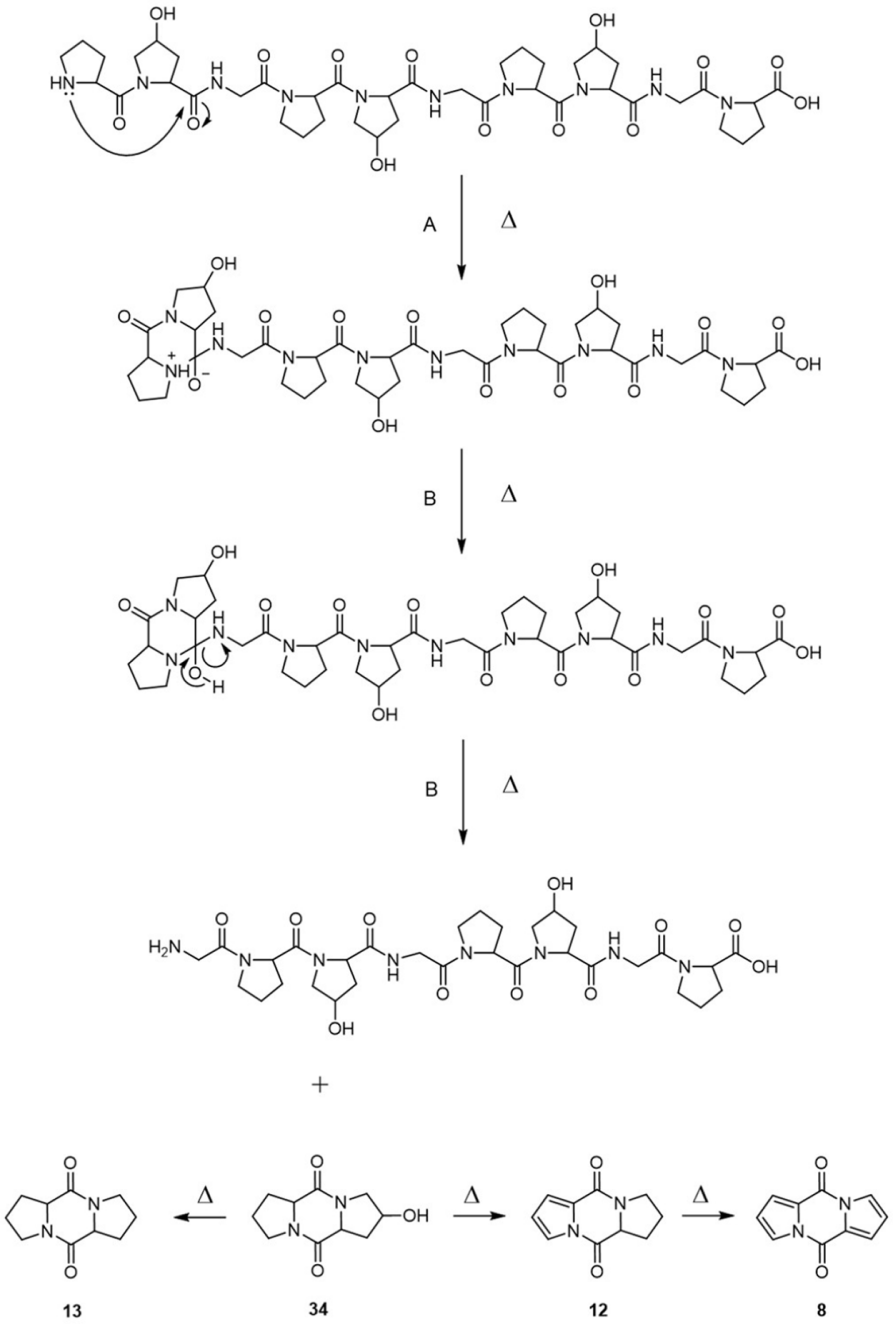
Example of a Collagen Peptide^58^ Forming DKPs as a Result of Pyrolysis^a^

a Cyclo­(Pro-Hyp) (34) forms from the neighboring Pro and Hyp residues. Further reactions may result in cyclo­(Pro-Pro) (13), cyclo­(Pro-Pyr) (12), and diketodipyrrole (8).

Diketodipyrrole, or cyclo­(Pyr-Pyr), has been observed to originate from the pyrolysis of Hyp^52^ and of Hyp-Hyp.^53^ It has been identified in considerable amounts in archeological bones,^21,50,54^ as well as in fossil bones^55^ and egg shells,^56^ and thus, it has been suggested as a collagen pyrolysis marker. Nonetheless, other pathways of formation were noted in the literature. These include the dimerization of Pro-Pro (or of Pro-Hyp and Hyp-Hyp),^50^ the elimination of dihydrogen from cyclo­(Pro-Pro), or even the pyrolysis of Pro-Arg^22^ (where it is believed to arise from the interaction of Pro residues).

Finally, because different products may result from the pyrolysis of free AAs and peptides, van Bergen et al. (1998) proposed that diketodipyrrole may also be formed from more complex reactions, involving peptides with different AA combinations.^57^ In any case, Hyp is largely responsible for diketodipyrrole formation, as cyclo­(Hyp-Hyp), expected to be present in considerable abundance, is not observed.

In contrast to animal glues, casein (aged spread, C1) yielded complex pyrograms, irrespective of whether the analysis was carried out with (Figure S2) or without TMAH (Figure 1b). Although better chromatographic resolution was sometimes achieved with TMAH analysis, this varied depending on how much sample was placed in the TSP, which is a difficult parameter to control. As was the case for animal glue, the generated DKPs from casein reflect the main AA composition of the protein, in addition to their likelihood of reaction on the basis of polarity and proximity. The obtained DIP-GC-MS TIC (without TMAH) was similar to published results for Py-GC-MS.^21^ The peak with the highest relative intensity corresponds to cyclo­(Pro-Pro) (peak 13), while nearby isomers of cyclo­(Pro-Val) (peaks 51 and 52) and cyclo­(Pro-Ile) (peaks 53, 54, and 55) are present. These peaks are not well separated, especially for cyclo­(Pro-Ile), which coelutes with cyclo­(Pro-Pro). Cyclo­(Pro-Ala) and diketodipyrrole are present at low relative intensity, in contrast to cyclo­(Pro-pyroGlu) (peak 56) and cyclo­(Pro-Phe) (present as two isomers, peaks 57 and 58), which elute in the latter portion of the TIC. The structure of cyclo­(Pro-pyroGlu), the dehydrated form of cyclo­(Pro-Glu), was confirmed by Fabbri et al. (2012) through ESI fragmentation and NMR studies^22^

The DIP-GC-MS TIC obtained from an aged egg white spread (EW1) presented considerably more pyrolysis products when analyzed with TMAH (Figure S3) than when analyzed without (Figure 1c). However, DKPs were observed only in the TIC resulting from the latter, albeit in low amounts. This TIC was dominated by propane nitrile (peak 4), benzyl nitrile (peak 5), indole (peak 5, but coeluting with (E)-3-phenylpropenenitrile, peak 62), and 2-cyano-benzoic acid (peak 63). This contrasts with published results from Orsini et al. (2017), who, from Py-GC-MS of egg albumin at 350 °C, obtained a TIC characterized by a high amount of cyclo­(Pro-Leu), cyclo­(Pro-Ile), and cyclo­(Pro-Val).^21^ Instead, in the present analysis, only cyclo­(Pro-Ile) (peaks 53 and 54) and diketodipyrrole (peak 8) were observed in low amounts. Finally, hexadecanenitrile (peak 64) and octadecanenitrile (peak 65), used as markers for the presence of egg^59^ in aged paintings, may be observed.

The DIP-GC-MS TICs obtained for the animal glues are distinctive from those of the other proteinaceous binders. They are characterized by only a few, well-separated, peaks, the most dominant of which are related to distinctive DKPs. Minimizing the risk of coelution and consequential interferences in isotopic determinations, this profile is suitable for targeting DKPs with DIP-GC-C-IRMS analysis. Conversely, egg white and casein present more complex TICs, with numerous pyrolytic products eluting close together and a similar DKP profile. Egg white, in particular, displays a higher abundance of nonspecific markers and a low abundance of DKPs. For these materials, the poorer baseline resolution heightens the risk of undetected coeluting peaks during DIP-GC-C-IRMS, rendering them less amenable to this type of isotopic analysis. Additionally, their isotopic signatures are not expected to be particularly distinctive.

### Bulk Stable Carbon and Nitrogen Isotope Analysis of Protein References

Although bulk stable isotope analysis is unsuitable for application to painting samples, due to their intrinsic heterogeneity and contaminated nature, it is an extremely useful tool to gain an initial assessment of isotopic signatures of reference materials. The bulk δ^13^C and δ^15^N compositions of uncontaminated, modern protein references for duck egg (EWD and EYC), chicken egg (EWC and EYC), casein (C2), and rabbit skin (GRS1 and GRS2) and isinglass (GI1 and GI2) glues were obtained through EA-IRMS. The individual values are plotted in Figure 2 and listed in Table S2. Of the isinglass glues, GI2 is known to derive from sturgeon, while GI1 is presumed to derive from sturgeon, being the traditional fish used for isinglass production.^9^ Although some species of sturgeon exclusively inhabit freshwater environments, GI1 and GI2 exhibit a notably less depleted isotopic signature, typical of marine ecosystems.

**Figure 2:**
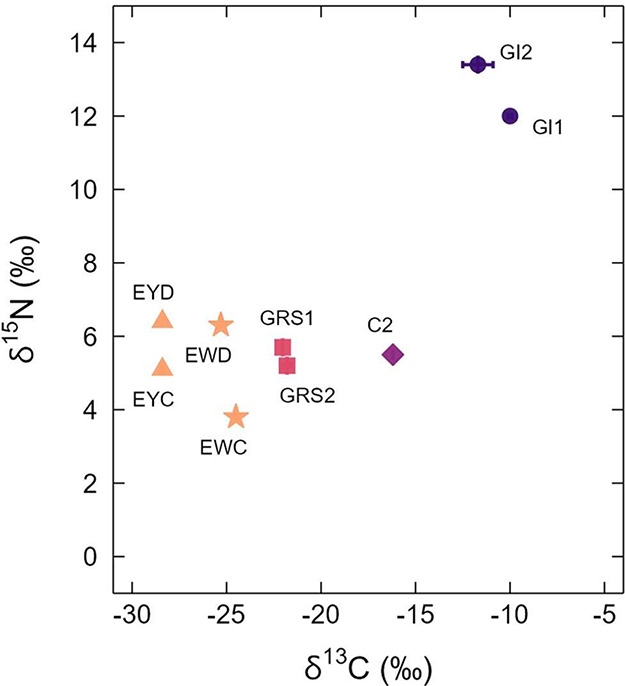
Bulk δ^15^N against bulk δ^13^C values for isinglass (GI1 and GI2, blue circles) and rabbit skin (GRS1 and GRS2, pink squares) glues, chicken (EWC) and duck (EWD) egg albumins (orange stars) and yolks (EYC and EYD) (orange triangles), and casein (C2, purple diamond) references. The error bars represent the SD on either side (±) of the value.

As illustrated by Figure 2, isinglass glues may be clearly distinguished not only from rabbit skin glues but also from other proteins. Specifically, the fish glues are, on average, more enriched by 7.4‰ (±0.3) in ^15^N and by 8.2‰ (±0.6) in ^13^C than the terrestrial glues. The latter plot close together, exhibiting δ^13^C values typical of a C_3_-based diet (−22.0‰ ±0.02 for GRS1 and −21.8‰ ±0.2 for GRS2). Interestingly, the duck egg albumin is more enriched (by 2.5‰ ±0.04) in ^15^N than the chicken egg albumin, while their δ^13^C values are similar (−24.5‰ ±0.1 for EWC and −25.3‰ ±0.1 for EWD). This trend is reflected in the egg yolks, although there is a less pronounced depletion in ^15^N for chicken (5.1‰ ±0.1) compared to duck (6.4‰ ±0.1), while δ^13^C values are identical (−28.4‰). Finally, the δ^13^C value for C2 (−16.2‰ ±0.04) reveals that the animal from which the protein originates consumed a predominantly C_4_-based diet. This is consistent with previous analysis by Corr (2003), who investigated the same material.^30^

### Compound-Specific δ^**13**^C Value Determination of AAs

Due to the considerable difference in ^13^C depletion between aquatic and terrestrial animal collagen, determining DKP δ^13^C (δ^13^C_DKP_) values of these materials would enable distinction between fish and terrestrial animal glues in painting samples. As far as the authors are aware, no published work exists on δ^13^C value determination of DKPs. These compounds, originating directly from AAs, are expected to largely retain the isotopic signature of their precursors. Hence, δ^13^C values of single AAs (δ^13^C_AA_) in the protein references were determined through GC-C-IRMS to verify the relationship between DKPs and their parent AAs and to confirm the reliability of any DKP δ^13^C value determined in further analysis. After the proteins were hydrolyzed, AAs were prepared as NAIP ester derivatives to render them GC amenable. Compared to other methods, this option presents a reduced amount of added carbon, in addition to a good baseline and chromatographic separation afforded by these compounds.^37^

All determined δ^13^C_AA_ values for protein references are listed in Tables S3 and S4. As expected, δ^13^C_AA_ values of chicken and duck eggs did not differ enough to be utilized for separating sources, while those of casein were similarly indistinct and diet-dependent. The present discussion will thus be limited to δ^13^C_AA_ values relevant to DKP markers for animal glues, namely Ala, Gly, Pro, and Hyp. In general, as presumed, AAs from isinglass glues were found to be more ^13^C-enriched than those from rabbit skin glues.

As Hyp is derived from Pro, and no carbon bonds are broken during the reaction, no isotopic fractionation is expected, and the two AAs should exhibit the same δ^13^C values. For both GRS1 and GRS2, Pro δ^13^C values were determined to be −23.2‰ ±0.2, while for Hyp, these were −22.3‰ ±0.1 and −22.5‰ ±0.3, respectively. For GI1 and GI2, δ^13^C_Pro_ values were established to be −13.6‰ ±0.2 and −17.0‰ ±0.1, and δ^13^C_Hyp_ values −13.1‰ ±0.1 and −16.4‰ ±0.2, respectively. Although Hyp was slightly less depleted, the biggest difference from the corresponding δ^13^C_Pro_ value was minimal (0.6‰ ±0.2, for GRS1).

For GRS1 and GRS2, the δ^13^C_Ala_ value was nearly identical, measuring −20.8‰ ±0.3 and −20.9‰ ±0.2, respectively. In the isinglass glues, Ala was less depleted in GI1 (−12.0‰ ±0.3), than in GI2 (−15.6‰ ±0.2). For the fish glues, however, Gly, after Ser, was the most ^13^C-enriched of the investigated AA. The difference in depletion from Gly in the terrestrial glues was significantly greater than for the other AAs. The determined δ^13^C_Gly_ values for GI1 and GI2 were −3.7‰ ±0.3 and −9.4‰ ±0.2, respectively. These are notably less depleted than the terrestrial glues. Respectively, they are 15.8‰ ±0.4 and 10.1‰ ±0.3 more enriched than the δ^13^C_Gly_ value for GRS1 (−19.5‰ ±0.2), and 16.1‰ ±0.4 and 10.4‰ ±0.3 more enriched than the δ^13^C_Gly_ value for GRS2 (−19.8‰ ±0.2).

In Figure 3a, the δ^13^C_Gly_ values for the animal glues have been plotted against the δ^13^C_Pro_ values, highlighting the lower depletion exhibited by the isinglass glues. Associations between marine organisms and notably less negative δ^13^C_Gly_ values is a known phenomenon. Corr et al. (2005) demonstrated that the different metabolic pathways of Gly in terrestrial and marine collagen may be utilized to differentiate between consumers of these different diets through the Δ^13^C_Gly–Phe_ proxy.^31^ In the present study, as Phe is not found in animal glue DKP markers, the Δ^13^C_Gly–Pro_ proxy was utilized instead. As illustrated in Figure 3b, this value is just as successful at reflecting the distinct metabolic pathways of Gly in the terrestrial and marine glues, and the resulting different relationship between the same AAs.

**Figure 3:**
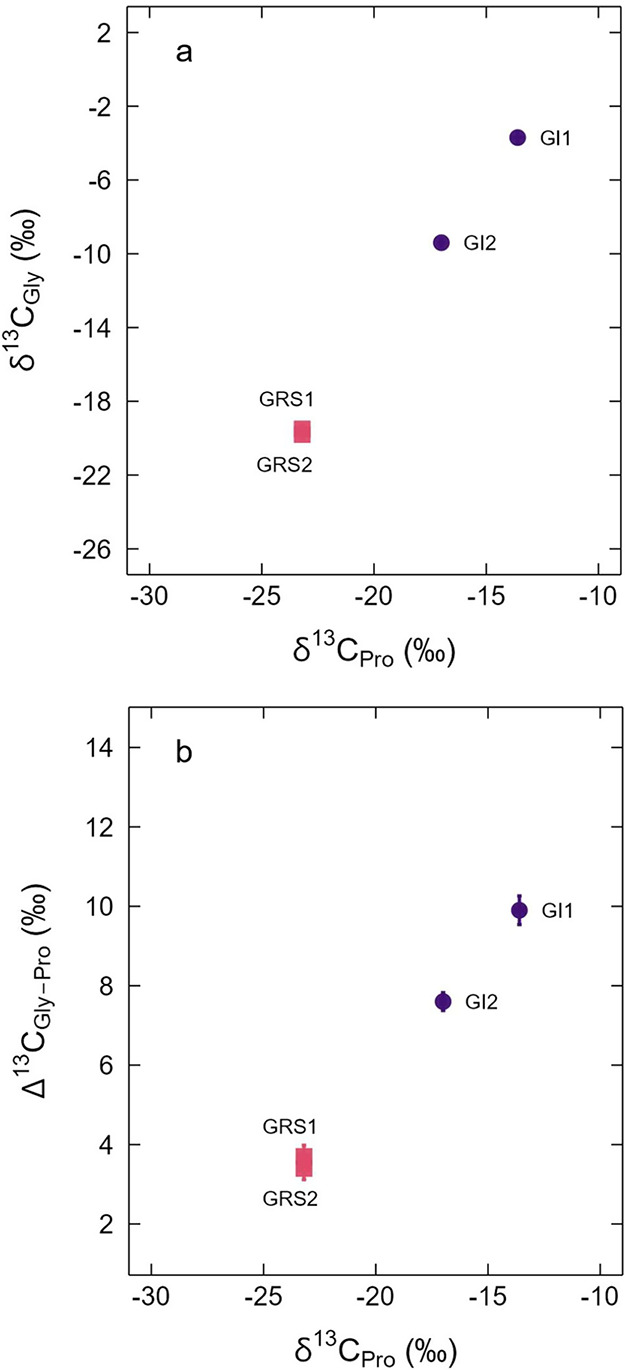
(a) δ^13^C_Gly_ values for isinglass (GI1 and GI2, blue circles) and rabbit skin (GRS1 and GRS2, pink squares) glues, plotted against their δ^13^C_Pro_ values. (b) Δ^13^C_Gly–Pro_ values, for the same glues, plotted against their δ^13^C_Pro_ values. The error bars represent the SD on either side (±) of the value. Instrumental precision was 0.3**‰**.

### Assessment of Robustness of Determined δ^13^C_DKP_ Values

As illustrated by Figure 4, analysis of the aforementioned glues by DIP-GC-C-IRMS produced chromatograms comparable to those obtained by DIP-GC-MS. Of the DKPs most typically found in animal glues, cyclo­(Pro-Gly) and cyclo­(Pro-Hyp), in particular, were found to exhibit sufficient peak baseline resolution to permit consistent δ^13^C_DKP_ value determinations. On the other hand, the peaks corresponding to cyclo­(Pro-Pro) and cyclo­(Pro-Pyr) display a lower relative intensity and poorer baseline separation. Given the consequential higher risk of interferences in isotopic determinations due to coelution, these DKPs were omitted from this investigation. Cyclo­(Pro-Ala) was similarly excluded, as it was identified as two isomers which coelute with unknown compounds. Even though one isomer of this DKP elutes extremely close to diketodipyrrole, the mass spectra of the two peaks do not highlight significant coelution (Figure 5a–c). For diketodipyrrole, this suggests enough baseline separation to be subjected to isotopic determinations by DIP-GC-C-IRMS. Thus, due to its prominence as a DKP marker for aged proteins, cyclo­(Pyr-Pyr) was included in this investigation alongside cyclo­(Pro-Gly) and cyclo­(Pro-Hyp).

**Figure 4:**
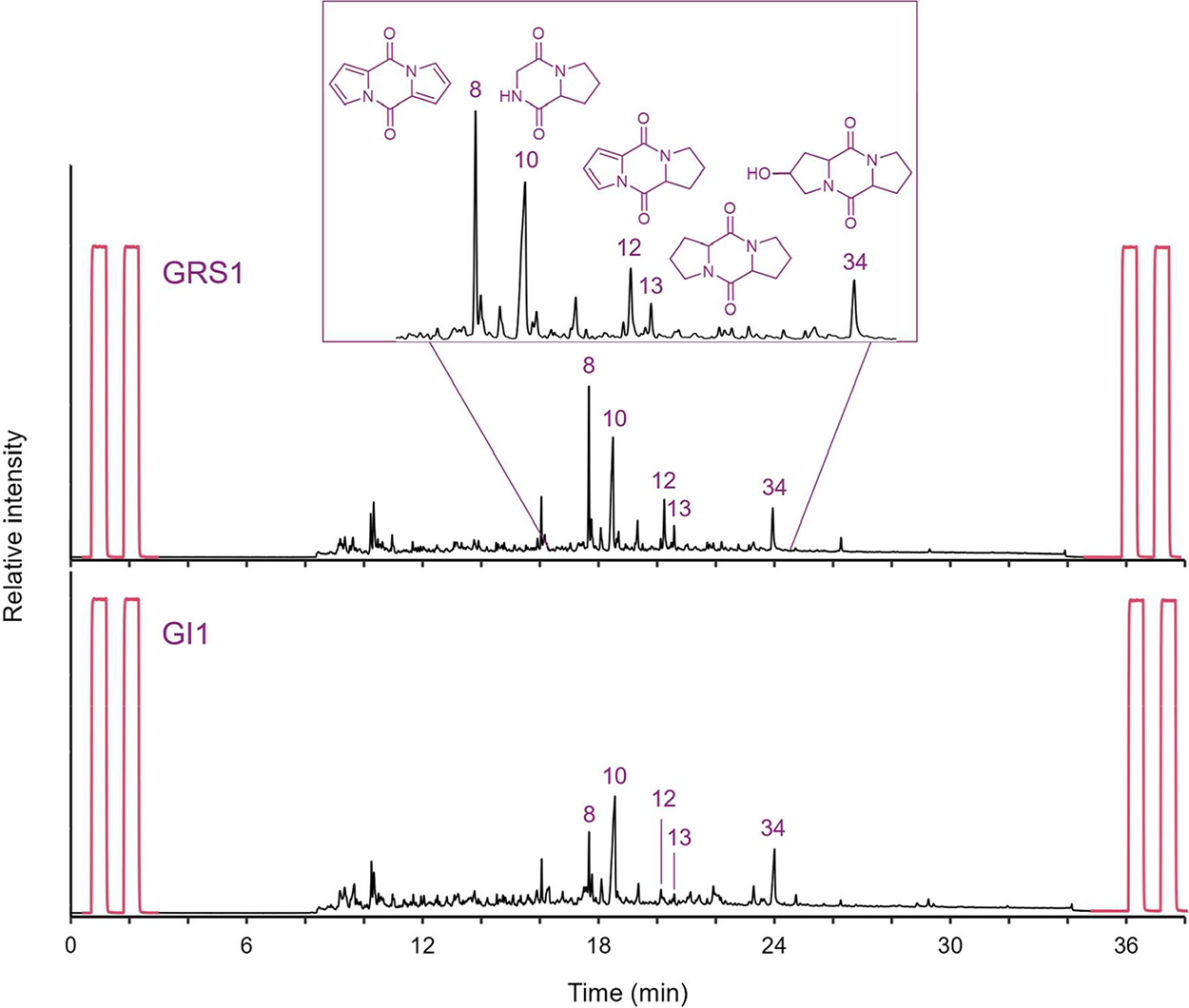
Typical DIP-GC-C-IRMS chromatograms, displaying the ion current signal for *m*/*z* 44, obtained from the analysis of rabbit skin glue, GRS1, and isinglass glue, GI1. For each glue, signals are shown at a common instrument-generated intensity scale. Relevant DKPs, with their structures, are indicated in purple in the enlarged area. Reference CO_2_ gas pulses, at the beginning and end of the chromatograms, are displayed in red. 8 = cyclo­(Pyr-Pyr), 10 = cyclo­(Pro-Gly), 12 = cyclo­(Pyr-Pro), 13 = cyclo­(Pro-Pro), 34 = cyclo­(Pro-Hyp).

**Figure 5:**
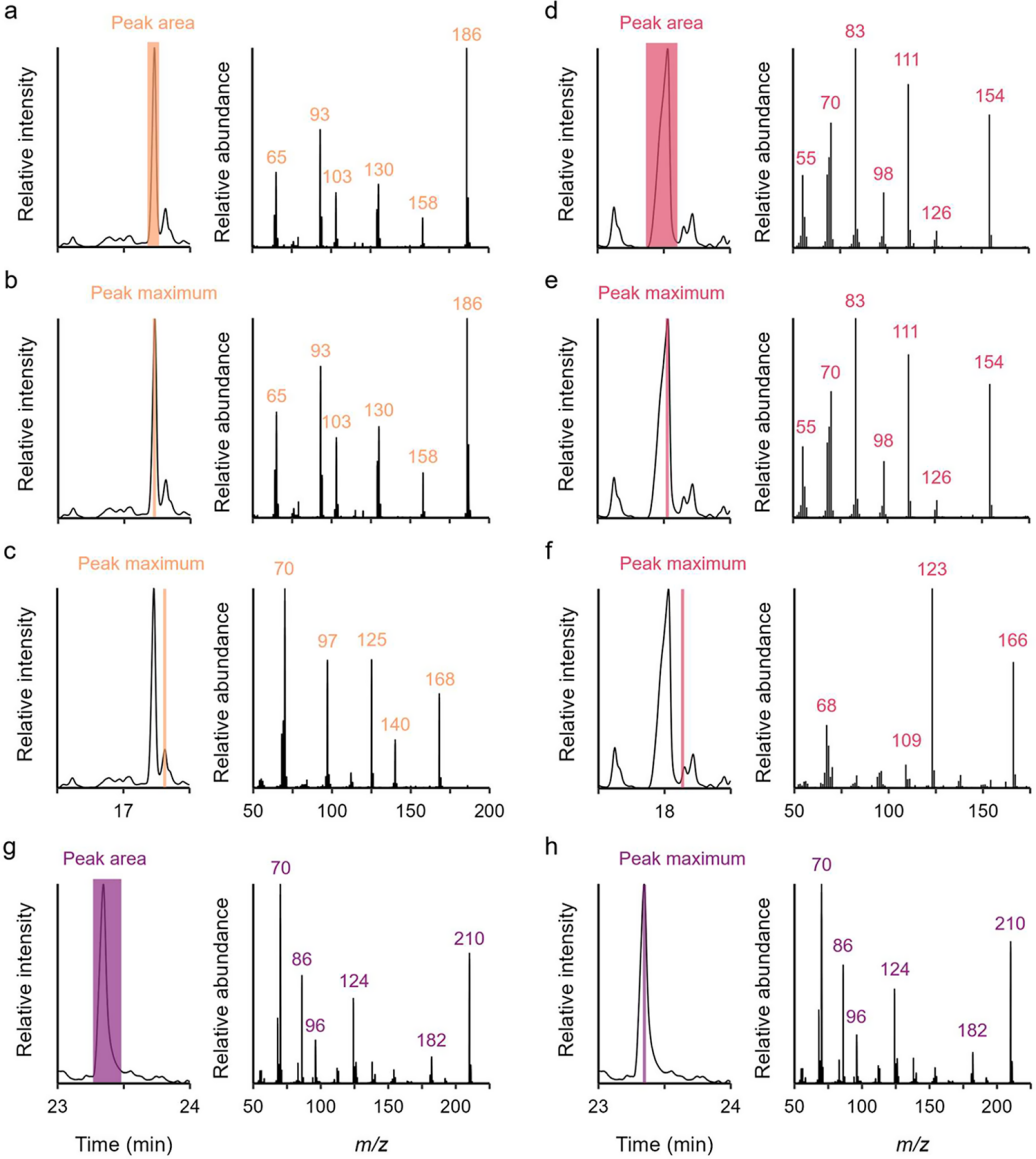
Partial DIP-GC-C-IRMS chromatograms of rabbit skin glue (GRS1), with relevant MS for (a,b) diketodipyrrole, (c) cyclo­(Pro-Ala), (d,e) cyclo­(Pro-Gly), (f) unknown compound eluting close to cyclo­(Pro-Gly), and (g,h) cyclo­(Pro-Hyp). For each DKP, MS of peak maxima are identical to entire peak areas, and MS of the closest eluting peaks (c,f) contain different ions, confirming that significant contamination is not occurring to affect the recorded δ^13^C values.

Previous studies have concluded that isotopic determinations are valid if the fractionation associated with the reaction is constant and reproducible.^60–62^ An assessment on any fractionation arising from the formation of DKPs was therefore carried out by performing DIP-GC-C-IRMS of GRS1 and GI2 multiple times (*n* = 7 and 6, respectively), yielding replicable chromatograms (see Figure S4). As δ^13^C_DKP_ value determination is undertaken on peaks arising from pyrolytic processes, poorer baseline separation and larger errors would then be acceptable for standard GC-C-IRMS methods, which were expected. Indeed, the SD ranged, typically, between 0.4 and 0.9‰, which, for some applications, may be a significant disadvantage. However, given the considerable difference observed in isotopic signatures between terrestrial and marine animal glues, the larger error was not deemed a limiting factor for the present study.

DIP-GC-C-IRMS analysis of animal glues was judged to yield reproducible δ^13^C_DKP_ values, demonstrating that any fractionation occurring during the reaction is consistent. Hence, this is a valid parameter to investigate isotopic composition. Similarly to the δ^13^C_AA_ values, δ^13^C_DKP_ values were more depleted for rabbit skin glue than for isinglass glue. The δ^13^C_Pro‑Hyp_ values were determined to be −13.3‰ ±0.4 and −19.8‰ ±0.9 for GI2 and GRS1, respectively. Reflecting the pattern seen for AAs, the glycine-containing DKP, cyclo­(Pro-Gly), was the least depleted, exhibiting δ^13^C values of −9.7‰ ±0.8 (GI2) and of −19.0‰ ±0.8 (GRS1). The δ^13^C_Pyr‑Pyr_ value of GI2, −11.7‰, displayed a notably large SD of 1.6‰. In this instance, the high variability in δ^13^C value may be due to the complexity of pathways leading to the formation of cyclo­(Pyr-Pyr), which may involve multiple combinations of dehydration and cyclization reactions, and the fact that it may originate from different amino acids.^22,50,57^ However, even taking into account the large error, this DKP was still considerably less depleted in GI2 than in GRS1, which exhibited a δ^13^C_Pyr‑Pyr_ value of −21.6‰ ±0.6.

DIP-GC-C-IRMS of the remaining glues, GRS2 and GI1, was undertaken in duplicate, as is standard practice in our laboratory. The analysis yielded reproducible δ^13^C_DKP_ values with SDs ranging between 0.2 and 0.9‰. Of all glues analyzed, δ^13^C_DKP_ values of GI1 exhibited the lowest SDs, of 0.2, 0.5, and 0.2‰, for δ^13^C_Pyr‑Pyr_, δ^13^C_Pro‑Gly_, and δ^13^C_Pro‑Hyp_ values, respectively. In addition to visual evidence from the chromatogram, a peak may be judged to be sufficiently separated to be accepted for isotopic determinations if it yields a reproducible δ^13^C value. Furthermore, to verify peak purity, rigorous examination of mass spectra (from DIP-GC-MS) was undertaken for all DKPs for which isotope values were recorded. In Figure 5a,d, the integrated peak area used to generate an isotope value for, respectively, cyclo­(Pyr-Pyr) and cyclo­(Pro-Gly), is highlighted. For each DKP, the corresponding mass spectrum for the entire peak area is identical to that of the peak maximum (Figure 5 b,e). In addition, it is devoid of ions present in the mass spectrum of the closest eluting peak on the right (Figure 5c,f), thus confirming that no coelution is occurring. Similarly, the peak purity of cyclo­(Pro-Hyp) was confirmed by comparing the mass spectrum of the entire peak area against the mass spectrum of the peak maximum (Figure 5g,h).

It should be noted that, whereas the rabbit skin glues were supplied as powder, the isinglass glues were supplied in the form of flakes. Variabilities in the samples, despite efforts to homogenize them before analysis, might have thus played a part in the larger errors observed for GI2. This is reflected in the EA-IRMS results, which reported a considerably larger SD (0.8‰) for the bulk δ^13^C value of this glue compared to all other proteins analyzed (for which max SD = 0.2‰). Although GI1 was supplied in thinner, smaller flakes than GI2, the latter was chosen for this assessment due to its more certain origin. Additionally, due to the pyrolytic nature of the analysis and microscopic quantities investigated, the amount of analyzed sample is harder to control than in liquid injection procedures. This is particularly relevant for reference pure material, where higher concentrations might result in a heightened baseline, requiring a repeat of the analysis. Finally, the method would be improved by using an analytical standard with compounds that more closely resemble the target analytes. If available, this might comprise a mixture of DKPs, analyzed by EA-IRMS to determine their δ^13^C value. Even in this case, however, DIP-GC-C-IRMS analysis of the standard will not fully mimic that of animal glue, given that, in the latter, DKPs are formed directly in the GC inlet.

### Assessment of Isotopic Fractionation during DKP Formation

The determined δ^13^C_DKP_ values, discussed above, for each animal glue are plotted in Figure 6 (listed in Table S5) together with the corresponding theoretical δ^13^C_DKP_ values (δ^13^C_DKPT_), calculated using weighted averages through the following equation 1:

$$\delta^{13}C_{\mathrm{DKPT}}=(\delta^{13}C_{\mathrm{AA}1}\times f_{1})+(\delta^{13}C_{\mathrm{AA}2}\times f_{2})$$ 1

where δ^13^C_AA1_ corresponds to the δ^13^C value of the first AA composing the DKP and f_1_ to the fraction of DKP carbon originating from AA_1_. The fractions (f_1_ and f_2_) of each AA in the DKP were calculated on the basis of contributing C atoms by dividing the number of C atoms in the DKP originating from AA_1_ (for f_1_) by the total number of C atoms in the DKP. The associated standard deviation σ_DKPT_ was calculated by propagation of uncertainties.^41^

**Figure 6:**
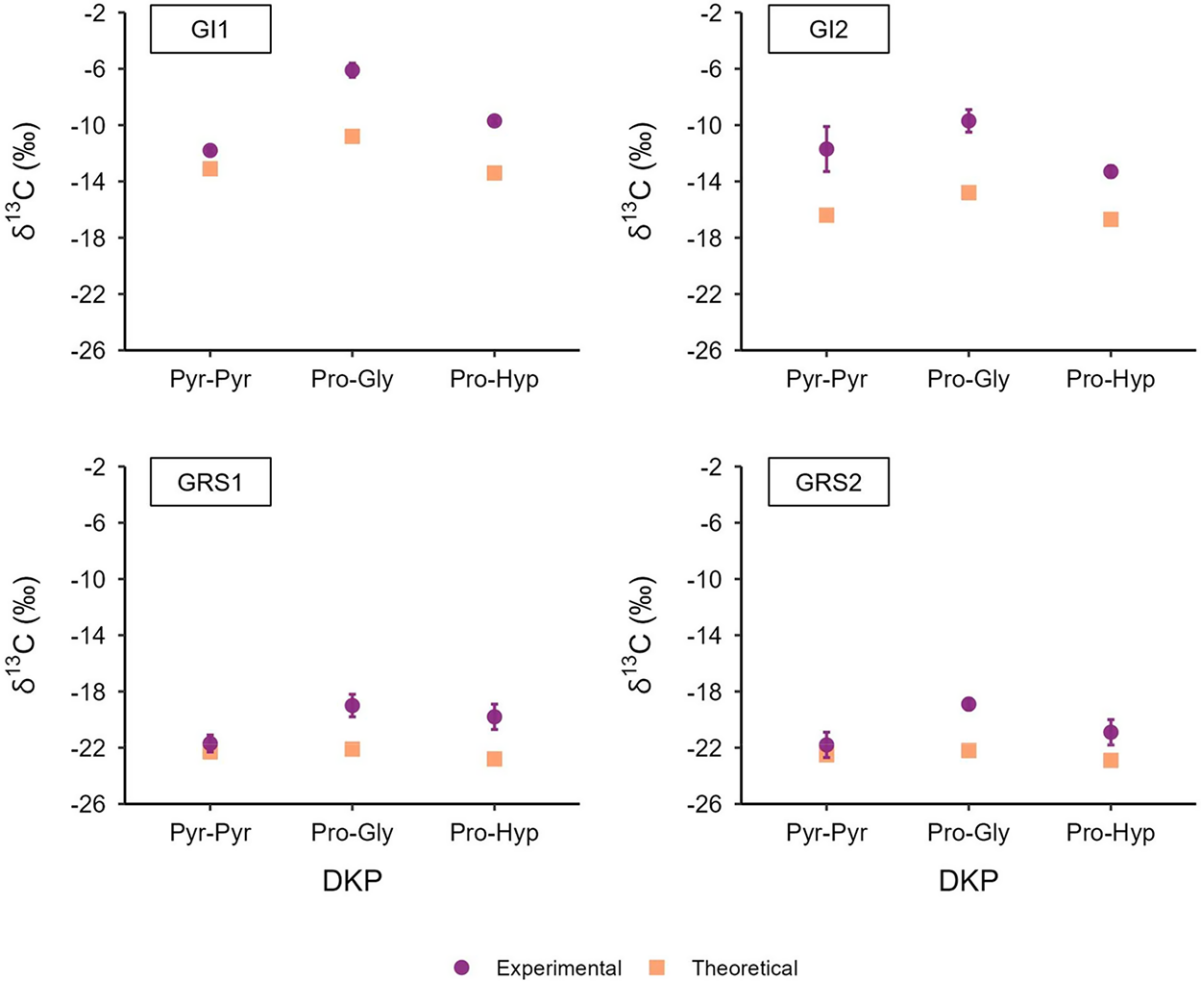
δ^13^C_DKP_ values obtained by DIP-GC-C-IRMS for rabbit skin (GRS1 and GRS2) and isinglass (GI1 and GI2) glues (experimental, purple circles), alongside the theoretical δ^13^C_DKPT_ values (orange squares). The error bars represent the SD on either side (±) of the value. Instrumental precision was 0.3**‰**.

Although diketodipyrrole may arise from various AA combinations, the theoretical δ^13^C_Pyr‑Pyr_ value was calculated assuming the DKP derived uniquely from Hyp. All δ^13^C_DKP_ values are enriched compared to the respective δ^13^C_DKPT_ values and follow the same pattern for each glue. On average, the enrichment for cyclo­(Pro-Gly) and for cyclo­(Pro-Hyp) was 4.0‰ (±1.0) and 3.0‰ (±0.7), respectively. Specifically, experimental δ^13^C_Pro‑Gly_ values were found to be between 3.1‰ (±0.8) and 5.1‰ (±0.8) greater than theoretical ones. The δ^13^C_Pro‑Hyp_ values exhibited a slightly more depleted range, between 2.0‰ (±0.9) and 3.6‰ (±0.2). For diketodipyrrole, a smaller difference between δ^13^C_DKPT_ and δ^13^C_DKP_ values was noted (1.8‰ ±1.9). However, given the wide SD and the possible different parent AA combinations, it is not possible to draw any conclusions from this figure.

The pyrolysis process does not cause isotopic fractionation,^63^ if no derivatization occurs. Thus, the results suggest that the cyclization of AA residues in collagen is accompanied by a kinetic isotope effect (KIE), promoting an enrichment in ^13^C in the formed DKPs. As the fractionation associated with this process was shown to be consistent and reproducible, the occurrence of KIEs does not affect the accuracy of the determination or interpretation of DKP δ^13^C values. Additionally, after analysis of a wider pool of reference animal glues, a calibration could potentially be introduced to reduce the effect of fractionation and to permit elucidation of AA δ^13^C values.

Primary KIEs cause the most significant isotopic fractionation. These may arise from a change in the bonds of the atoms involved in the rate-determining step, most commonly through bond breaking.^30,60^ AA standards of known isotopic composition were analyzed in pairs by DIP-GC-C-IRMS to investigate whether DKPs from free AAs would form under the same conditions and exhibit the same fractionation. The simpler reaction, devoid of unknowns such as precise AA linkages and configuration, might enable better understanding of any observed fractionation. Unfortunately, however, the analysis was inconsistent or unsuccessful for most of the AA combinations trialed. Free Gly and Pro, in particular, rarely yielded cyclo­(Pro-Gly), so it was not possible to determine δ^13^C_Pro‑Gly_ values from free AAs. This is believed to be due, at least partially, to the lower concentration and reduced proximity of the AAs in the inlet compared to pyrolysis of the protein. Compared to the strict configuration of animal glue proteins and peptides, free AAs have less opportunity to undergo condensation reactions. This is consistent with previous studies, which found that the formation of DKPs is governed by many factors, such as the steric and electronic nature of the substituents.^64^

Most of the AA combinations analyzed included Pro, and, as a result, diketodipyrrole was the most commonly formed DKP. However, only the analysis of Pro alone resulted in sufficiently abundant and well-resolved peaks to determine δ^13^C_Pyr‑Pyr_ and δ^13^C_Pro‑Pro_ values. These were found to not be significantly different from the known δ^13^C value for Pro (δ^13^C_Pyr‑Pyr_ – δ^13^C_Pro_ = – 0.5‰ ±0.2 and δ^13^C_Pro‑Pro_ – δ^13^C_Pro_ = 0.5‰ ±0.4; *n* = 2, instrumental precision = 0.3‰), suggesting that no or negligible isotopic fractionation is occurring during formation of these DKPs. Although direct comparison of δ^13^C values for the same DKP obtained from free AAs and from animal glues is not possible, some preliminary observations on DKP formation may be made. Despite the different AAs involved (Pro and Pro for free AAs, and Pro and Gly or Pro and Hyp for animal glues), the reaction begins with the attack of the amino group of one AA (or AA residue in proteins) on the carbonyl carbon of a neighboring AA or AA residue (see Scheme 1 for proteins and Scheme 2 for free AAs).

**Scheme 2:**
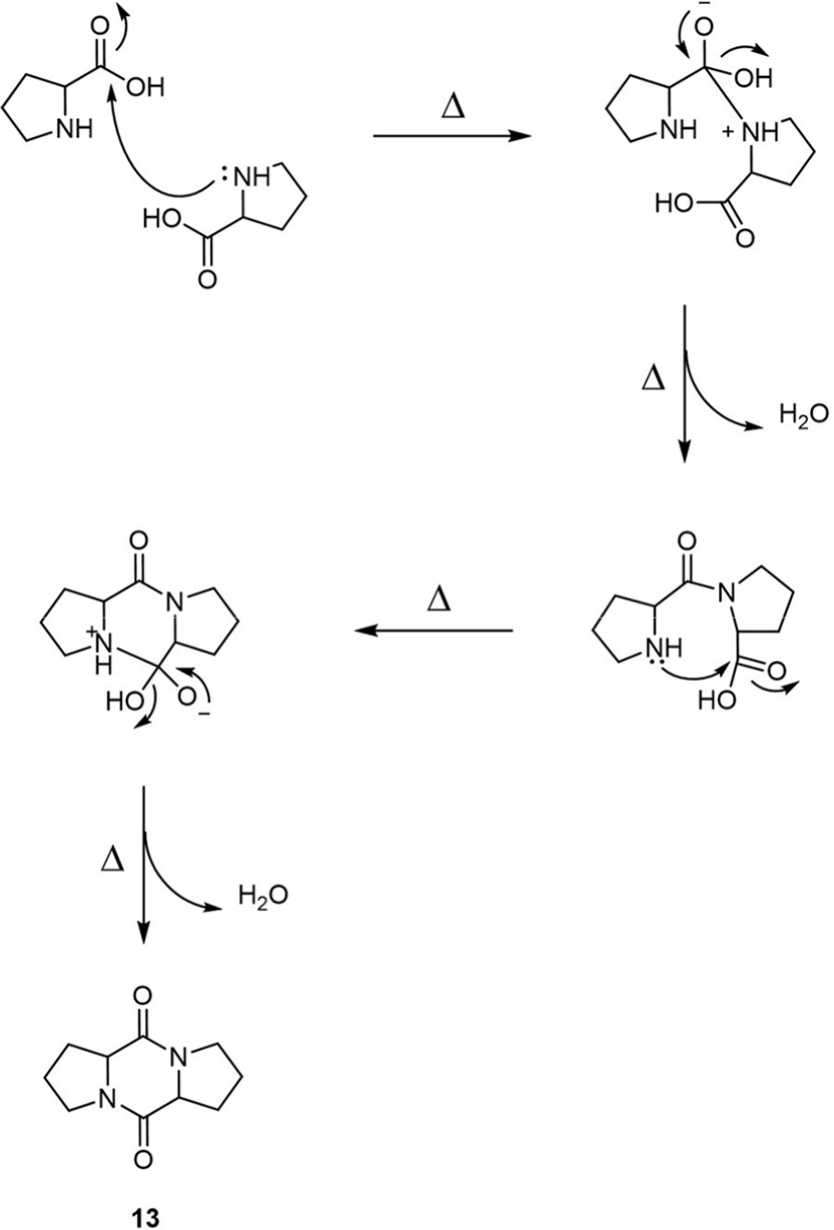
Formation of the DKP cyclo­(Pro-Pro) (13) from the Cyclization of the Free AA Proline

Although this step, involving the formation of a new C bond, could induce isotopic fractionation,^65^ this is less common than for bond breaking. In addition, if this were the rate-determining step, a more similar isotopic fractionation would be expected in DKPs formed from free AAs and from animal glues. The other, more likely, fractionation-inducing step is the conversion of the intermediate to the DKP. This step includes breaking of a C bond and differs between free AAs and proteins. In the case of free AAs, although a C–O bond is broken, this results in the loss of H_2_O. Being an energetically favored step, it is thus unlikely to result in incomplete conversion of the C-bond-containing reactant. This is a necessary requirement for an isotope effect to be observed.^30,60^ In proteins, conversely, either a C–C or C–N bond is broken, resulting, rather than in H_2_O loss, in the cleavage of the DKP from the peptide chain (step B to C in Figure 1). It is therefore likely that it is this step that induces isotopic fractionation in DKP formation in polypeptides and thus that it is rate-determining. Finally, this would agree with published studies on the reaction rate of DKP formation from H-Ala-Pro-NH_2_, which determined that the rate-determining step is the one from the intermediate to products.^66,67^

### Differentiation of Terrestrial and Marine Animal Glues through δ^13^C_DKP_ Values

By plotting δ^13^C_Pro‑Gly_ values against δ^13^C_Pro‑Hyp_ values obtained from DIP-GC-C-IRMS analysis of the reference animal glues (Figure S5a), a similar graph to that portrayed in Figure 3a is obtained. Thus, δ^13^C_DKP_ values, which parallel the isotopic imprint of their parent AAs, clearly differentiate terrestrial adhesives from marine ones. By using a proxy similar to that employed in Figure 3b, we obtain a potential additional tool for distinguishing between glue types. The new proxy Δ^13^C_(Pro‑Gly)–(Pro‑Hyp)_, denoting the difference in δ^13^C_Pro‑Gly_ and δ^13^C_Pro‑Hyp_ values, when plotted against the δ^13^C_Pro‑Gly_ values, highlights the distinct relationships that exist between the same AAs in the animal glue, according to whether they have a terrestrial or marine origin (Figure S5b).

### DIP-GC-C-IRMS for Source Determination of Proteins in Paintings from The National Gallery

Ground layer samples from two Italian Renaissance paintings in the National Gallery collection, NG599 (Madonna of the Meadows) and NG6368 (Tanaquil), were analyzed by GC by White in the 1980s.^15^ The author reported AA profiles consistent with the presence of animal glues, which, for this purpose, would be expected to be of terrestrial origin. However, given the low proportion of Pro and Hyp in NG6368, White discussed a possible aquatic origin for this glue. Analysis was repeated with the aforementioned DIP techniques, revealing very similar profiles to those of the reference animal glues (Figure S6). The protein binder in the ground layers of both paintings exhibited similar DKP stable carbon isotope signatures. In particular, δ^13^C_Pro‑Gly_ values were determined to be −18.4‰ (±0.1) and −17.6‰ (±0.1) for NG599 and NG6368, respectively. The δ^13^C_Pro‑Hyp_ values were found to equal −19.8‰ (±0.4) and −18.6‰ (±0.001), respectively.

It should be noted that the SD observed for these determinations is lower than what was reported for some of the reference binders discussed above. These errors are in line with those observed for standard GC-C-IRMS methods. As these paint fragments were in the form of very fine powder, typical of paint samples, these results support that sample inhomogeneities might be primarily responsible for wide SDs, rather than the DIP-GC-C-IRMS method itself. In the case of marine animal glues, more enriched δ^13^C_DKP_ values would be expected than those observed here, especially for cyclo­(Pro-Gly). Instead, the determined δ^13^C_DKP_ values suggest a terrestrial origin for the glues in both paintings. Indeed, by plotting the values as described above, as shown in Figure 7, both paintings fall extremely close to the reference rabbit skin glues. Hence, for NG599, these results are in agreement with the previous analysis by White (1984). However, regarding the origin of the binder from NG6368, these findings support a similar terrestrial origin, suggesting that a lower-than-average concentration of Pro and Hyp is not characteristic of fish glues.

**Figure 7:**
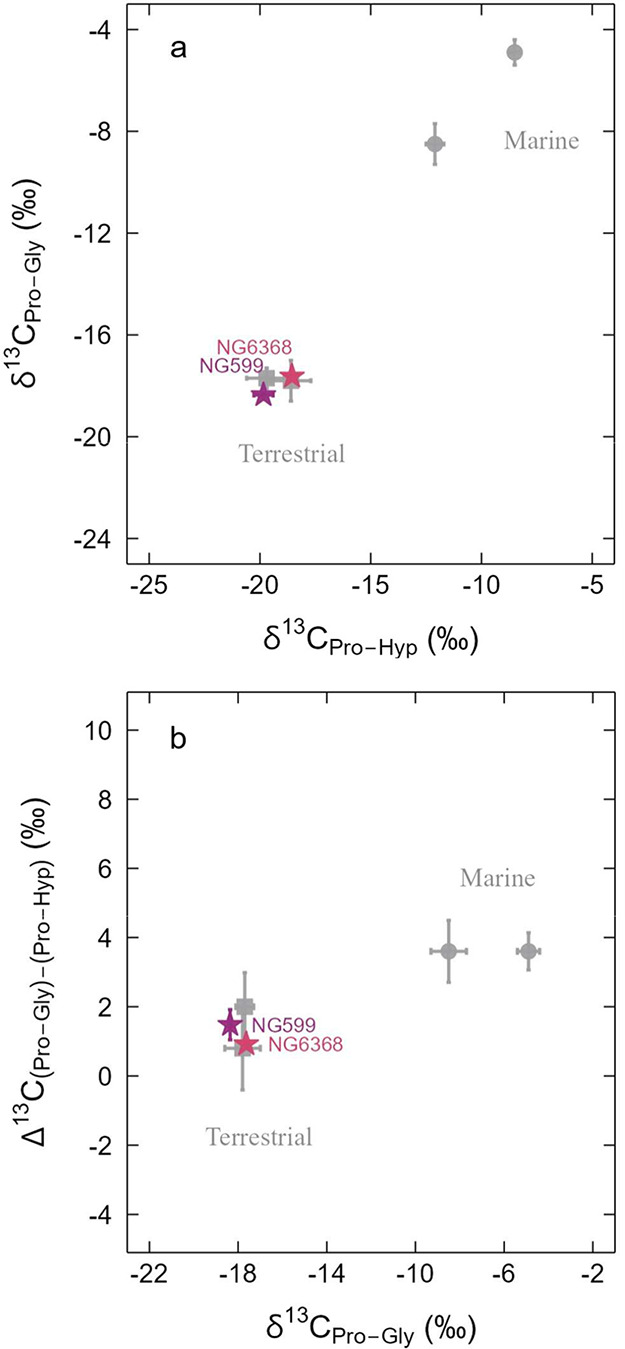
(a) δ^13^C_Pro‑Gly_ values for the ground layers from paintings NG599 (purple star) and NG6368 (pink star), against their δ^13^C_Pro‑Hyp_ values. Modern references for marine (GI1 and GI2) and terrestrial (GRS1 and GRS2) animal glues are in gray. (b) Corresponding Δ^13^C_(Pro‑Gly)–(Pro‑Hyp)_ values against their δ^13^C_Pro‑Gly_ values. The error bars represent the SD on either side (±) of the value. The δ^13^C values have been corrected to take into account post-Industrial Revolution effects of burning fossil fuels by the addition of 1.2‰.^68^ Instrumental precision was 0.3‰.

Although a wider range of reference material needs to be analyzed before this method may be reliably used, these results indicate that DIP-GC-C-IRMS is able to differentiate between terrestrial and marine animal glues in painting samples. Given the challenges currently faced by this field to differentiate fish glues from other animal glues, this innovative technique might enable new information to be gathered on artist techniques and the history of use of these materials. It is important to note that the method can be applied to many other fields of research. In addition to painting analysis, this minimally invasive protocol is pertinent to any discipline that involves the analysis of degraded proteins, heterogeneous and complex matrices, or where access to samples is restricted due to ethical implications or lack of material. This includes environmental and forensic sciences, paleo- and zooarcheology, paleoecology, and anthropology.

## Conclusions

Aged proteins encountered in painted cultural heritage have usually undergone extreme structural changes from the freshly applied material, often rendering unambiguous protein source determination unattainable. In particular, the distinction between terrestrial and aquatic animal glues is typically not possible without proteomics approaches. Nonetheless, this paper demonstrates that, with minimal sample sizes, DIP-GC-C-IRMS permits determination of δ^13^C values of DKPs arising from pyrolysis of aged proteinaceous material, providing the first record of the isotopic signature of these compounds. In this study, δ^13^C values of DKPs were shown to reflect the δ^13^C values of the parent AAs and, therefore, to permit unambiguous differentiation between terrestrial and marine animal glues, analogous to the use of CSIA of archeological collagen for palaeodietary reconstruction.

Remarkably, DIP-GC-C-IRMS, requiring a considerably smaller sample (in the microgram range) than typical GC-C-IRMS methods (in the milligram range), renders CSIA accessible for the investigation of proteinaceous material in painted cultural heritage for the first time. This widens the application of CSIA to precious, unique, and fragile objects in cultural heritage and beyond. Moreover, the rapidity of the analysis places DIP-GC-C-IRMS as a useful screening method to identify materials of interest for further proteomic analyses.

First, the isotopic imprint of modern references for rabbit skin glues, isinglass glues, casein, chicken, and duck eggs was confirmed through established EA-IRMS and GC-C-IRMS methods. As expected, bulk and individual AA δ^13^C and δ^15^N values for terrestrial animal glues exhibited a higher depletion in ^13^C and ^15^N than the marine glues. The δ^13^C values of DKPs arising from DIP-GC-C-IRMS of the same materials were found to retain this relationship. Furthermore, the ground layers of two 16th-century Italian paintings from the National Gallery collection, known to contain animal glue, yielded δ^13^C_DKP_ values consistent with the terrestrial variety, confirming that the isotopic signature of these compounds is retained through aging and degradation.

## Supplementary Material



## Notes

Instrument methods (S1) and EA-IRMS, DIP-GC-MS, and (DIP-)­GC-C-IRMS data (S2) (PDF)

## §

Conservation & Science, The Art Institute of Chicago, 111 S. Michigan Ave., Chicago, Illinois 60603, United States
