# Supplementary material for: Pyrolysis Compound-Specific Isotope Analysis of Proteins in Aged Paintings: Diketopiperazine δ13C Values as Collagen Source Indicators
Source: Anal Chem. 2026 Jul 15;98(29):21183–96. doi: 10.1021/acs.analchem.5c07901 (PMC13425550; doi:10.1021/acs.analchem.5c07901)
Supplement: Supplementary file 1 [file ac5c07901_si_001.pdf]

## Supporting Information

# Pyrolysis compound-specific isotope analysis of proteins in aged paintings: diketopiperazine $\delta^{13}C$ values as collagen source indicators

Eugenia Geddes da Filicaia*[1,2†], David A. Peggie[2], Iain P. Kendall[1], Ian D. Bull[1], Richard P. Evershed[1]

[1]Organic Geochemistry Unit, School of Chemistry, University of Bristol, Bristol BS8 1TS, U.K.;
[2]Scientific department, The National Gallery, Trafalgar Square, London WC2N 5DN, U.K.
†Present address: Conservation & Science, The Art Institute of Chicago, 111 S Michigan Ave, Chicago, IL 60603, U.S.A.

Corresponding author: email e.geddesdafilicaia@bristol.ac.uk

**Table of contents**

S1 Instrument methods ..... 1
S2 Additional EA-IRMS, DIP-GC-MS, and (DIP-)GC-C-IRMS data ..... 3
References ..... 11

**S1 Instrument methods**

**EA-IRMS.** Bulk $\delta^{13}C$ and $\delta^{15}N$ analysis was carried out using an Elementar IsoPrime PrecisION continuous flow isotope ratio mass spectrometer coupled with an Elementar vario PYRO cube elemental analyzer (EA) inlet (Elementar Analysensysteme GmbH, Hanau, Germany). The EA was calibrated with sulfanilamide. (N: 16.26%, C: 41.81%, S: 18.62%) and the precision as a relative standard deviation (RSD) was < 5% for both C and N. The IRMS was calibrated against international reference caffeine standards: USGS61 ($\delta^{13}C$ = –35.05‰, $\delta^{15}N$ = –2.87‰), USGS62 ($\delta^{13}C$ = –14.79 ‰, $\delta^{15}N$ = 20.17‰) and USGS63 ($\delta^{13}C$ = –1.17‰, $\delta^{15}N$ = 37.83‰). The precision as a standard deviation (SD) was < 0.06‰ for both $\delta^{15}N$ and $\delta^{13}C$ value determinations. Solid samples were weighed into Sn capsules before loading onto the instrument and analyzing in triplicate (quintuplicate for the reference materials, if enough sample was available). Liquid samples (25 µL) were manually injected into Sn capsules and measured in quintuplicate. Results were blank corrected.

**GC-FID.** Screening of NAIP derivatized AA hydrolyzed protein extracts was performed on an Agilent Technologies 7890B GC (Agilent Technologies, Santa Clara, CA, USA) equipped with a mid-polar fused silica capillary column (30 m × 0.32 mm i.d. × 0.50 µm film thickness) with a DB-35 stationary phase (35% phenyl methylpolysiloxane, Agilent Technologies). Extracts (1 µL) were injected into the MMI in split mode with an inlet temperature beginning at 75 °C (held for 0.1 min) and rising to 250 °C at a rate of 750 °C min–1, where it was held for 5 min before returning to 75 °C. The oven program began at a temperature of 70 °C, where it was held isothermally for 2 min, before being increased to 150 °C (at 15 °C $min^{-1}$), 210 °C (at 2 °C $min^{-1}$), and 270 °C (at 8 °C $min^{-1}$), where it was held for 10 min. He was used as carrier gas at constant flow (2 mL $min^{-1}$), and an FID, set to 300 °C, used to monitor column effluent. Data acquisition and processing were conducted using Agilent MSD ChemStation software (F.01.01.2317, Agilent Technologies). An in-house quality control standard containing AA NAIP esters, including Ala, Asp, Glu, Gly, Hyp, Ile, Leu, Lys, Nle, Phe, Pro, Ser, Thr, and Val was used to monitor instrument performance.

**GC-C-IRMS.** Analysis was carried out on an Agilent Technologies 7890B GC coupled to an Elementar isoprime precisION IRMS via an Elementar GC5 combustion interface, using a combustion/reduction reactor containing oxidised Cu and Ni wires (Elementar Analysensysteme GmbH, Hanau, Germany)**,** maintained at 950 °C. The GC was equipped with a mid-polar fused silica capillary column (30 m × 0.32 mm i.d. × 0.50 µm film thickness) with a DB-35 stationary phase (35% phenyl methylpolysiloxane, Agilent Technologies). Extracts (1 µL) were injected via the MMI in splitless mode with an inlet temperature beginning at 60 °C and rising to 270 °C at a rate of 900 °C $min^{-1}$ (held for 60 min). The oven program began at a temperature of 40 °C, where it was held isothermally for 2 min, before being increased to 120 °C (at 15 °C $min^{-1}$), 180 °C (at 3 °C $min^{-1}$), 210 °C (at 1.5 °C $min^{-1}$), and 270 °C (at 5 °C $min^{-1}$), where it was held for 1 min. He was used as carrier gas at constant flow (1.4 mL $min^{-1}$). The mass spectrometer was equipped with three Faraday cups collecting for the masses *m/z* 44 ($^{12}C^{16}O_2$), 45 ($^{13}C^{16}O_2$ and $^{12}C^{17}O^{16}O$), and 46 ($^{12}C^{18}O^{16}O$), with a solvent delay of 8 min. Data acquisition and processing were undertaken using the lyticOS software (Elementar, version 5.0.4.118). Reference $CO_2$ and in-house quality control standard containing FAMEs ($C_{11:0}$, $C_{13:0}$, $C_{16:0}$, $C_{18:0}$, $C_{21:0}$, and $C_{23:0}$) of known $\delta^{13}C$ values were used to monitor instrument performance. The FAME standard was also used for correction of determined $\delta^{13}C$ values using a two-point normalization. Calibration of $\delta^{13}C$ values due to the addition of C atoms from derivatization was undertaken using an AA NAIP esters standard of known AA $\delta^{13}C$ values (containing Ala, Asn, Glu, Gly, Hyp, Ile, Leu, Lys, Nle, Phe, Pro, Ser, Thr, and Val)[1]. The $\delta^{13}C$ values are reported relative to the reference $CO_2$, preliminarily calibrated against the VPBD international isotope standard[2]. This was introduced directly into the ion source in three pulses, at the beginning and end of each run[3]. All sample $\delta^{13}C$ values are a mean of duplicate analyses.

## S2 Additional EA-IRMS, DIP-GC-MS, and (DIP-)GC-C-IRMS data

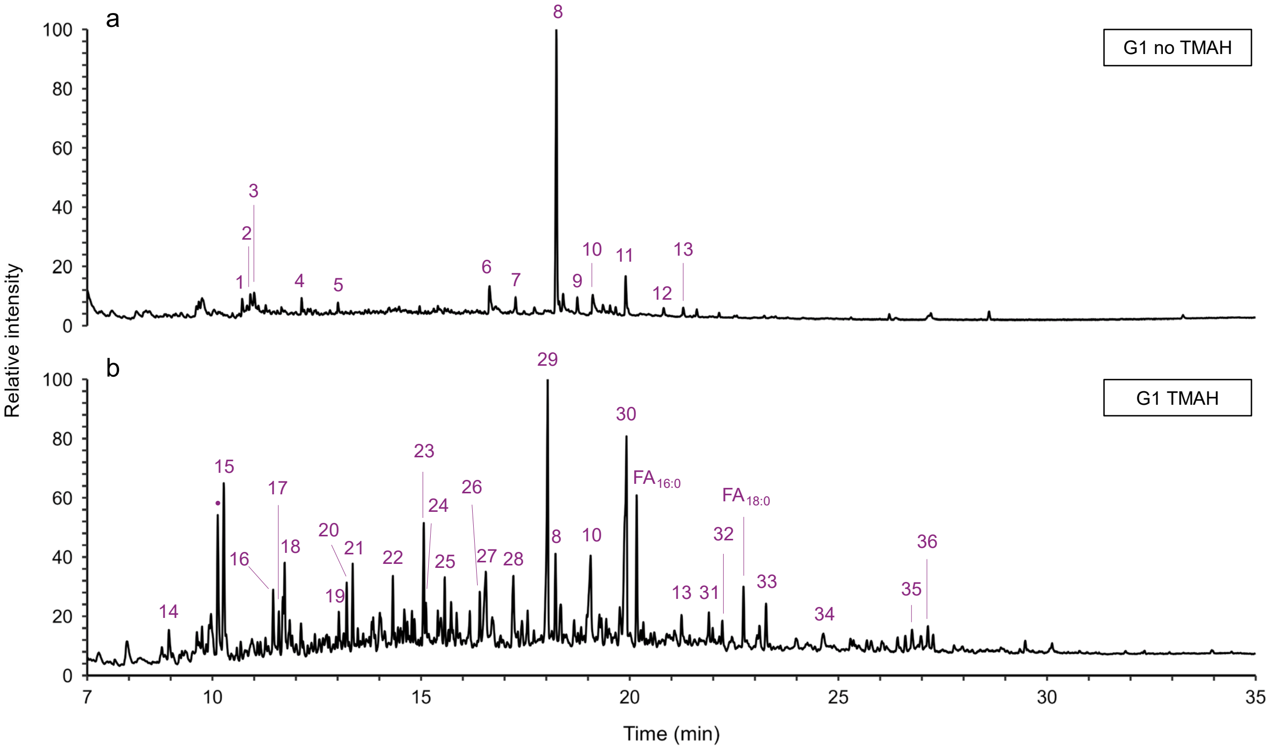


**Figure S1.** Partial GC-MS TIC obtained from the analysis of gelatine, G1, by DIP-GC-MS without (a) and with (b) TMAH. • indicates TMAH degradation byproducts. Information on numbered peaks is listed in Table S1.

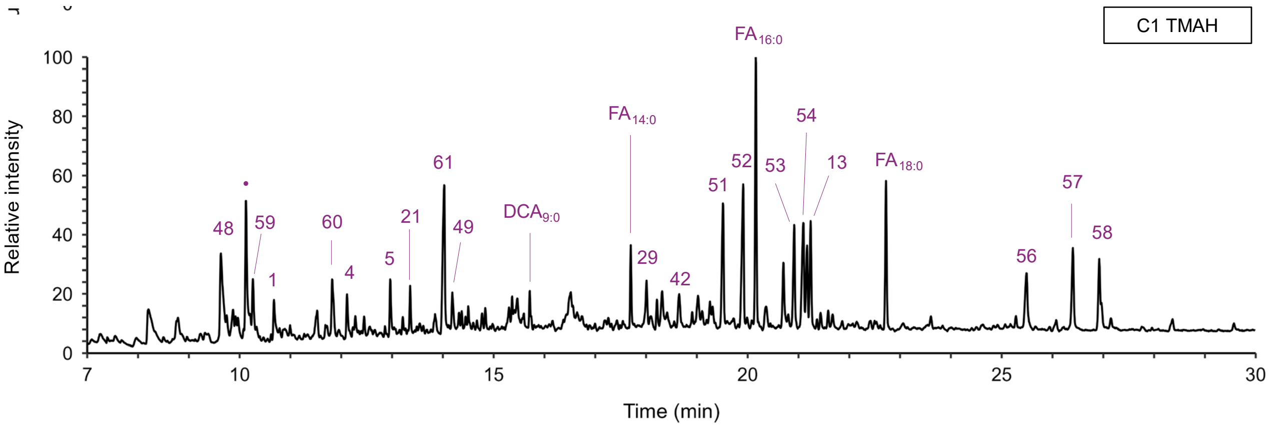


**Figure S2.** Partial GC-MS TIC obtained for the analysis of casein, C1, by DIP-GC-MS with TMAH. • indicates TMAH degradation byproducts. Information on numbered peaks is listed in Table S1.

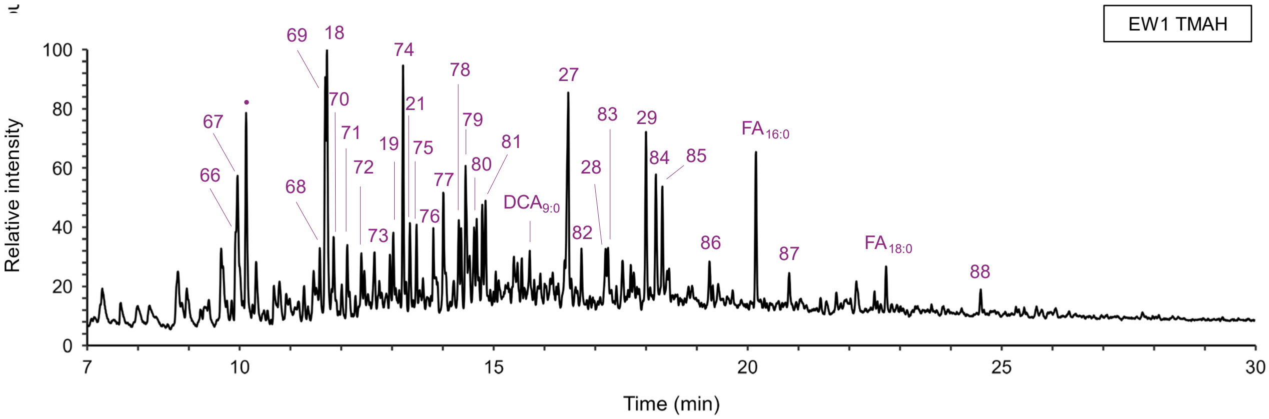


**Figure S3**. Partial GC-MS TIC obtained for the analysis of egg white, EW1, by DIP-GC-MS with TMAH. • indicates TMAH degradation byproducts. Information on numbered peaks is listed in Table S1.

**Table S1**. Peak number, compound, *m/z* (base peak in bold and molecular ion peak in parenthesis), and retention time for peaks for the analysis of gelatine (G1), isinglass glue (GI2), casein (C1), and egg white (EW1) by DIP-GC-MS or DIP-GC-QToF-MS* with and without TMAH.

| Peak | Compound | *m/z* | Retention time (min) |
|---|---|---|---|
| 1 | Benzyl nitrile | 90, (**117**) | 10.7 |
| 2 | 1H-Pyrrole-3-carbonitrile | 65, (**92**) | 10.9 |
| 3 | ? | 80, 93, **121**, 134 | 11.0 |
| 4 | Benzenepropanenitrile | **91**, (131) | 12.1 |
| 5 | Indole | 89, 90, (**117**) | 13.0 |
| 6 | ? | 80, **107**, 136 | 16.7 |
| 7 | 1,3-Diphenylpropane | **92**, 105, (196) | 17.3 |
| 8 | Diketodipyrrole | 65, 93, 130, (**186**) | 18.2 |
| 9 | ? | 70, 93, 171, **172**, 186 | 18.7 |
| 10 | Cyclo(Pro-Gly) | 83, 98, **111**, (154) | 19.1 |
| 11 | ? | 52, 79, 107, **200** | 19.9 |
| 12 | Cyclo(Pro-Pyr)* | 68, 70, **191**, 192 | 20.8 |
| 13 | Cyclo(Pro-Pro) | **70**, 96, 138, (194) | 21.3 |
| 14 | ? | **84**, 109, 125 | 9.0 |
| 15 | Methyl pyrrole-2-carboxylate | 66, 93, **94**, (125) | 10.3 |
| 16 | ? | 82, 99, 109, **114**, 127 | 11.5 |
| 17 | 1,3-Methyldihydro-2,4(1H,3H)-pyrimidinedione | 70, 99, 100, (**128**) | 11.6 |

| | | | |
|---|---|---|---|
| 18 | Protein marker (AMDIS) | 56, **127**, 142 | 11.7 |
| 19 | ? | **56**, 138 | 13.0 |
| 20 | ? | **108**, 128, 138 | 13.2 |
| 21 | 1-Methyl-5-oxo-l-proline methyl ester | 70, **98**, 157 | 13.4 |
| 22 | ? | **69**, 82, 114, 126, 154 | 14.3 |
| 23 | Protein marker (AMDIS) | 56, 71, **113**, 156 | 15.1 |
| 24 | N,N'-Cyclo(Gly-Gly) | 57, 85, 113, (**142**) | 15.1 |
| 25 | (i),6-Dimethyl-3-methylidenepiperazine-2,5-dione (i=1 or 4) derivative | 55, 125, **154**, 158, 167 | 15.6 |
| 26 | ? | 113, **142**, 168, 196 | 16.4 |
| 27 | 2,5-pyrrolidinedione, 1-methyl (derivative) | 70, **98**, 136, 156 | 16.5 |
| 28 | ? | 55, 113, **142**, 176 | 17.2 |
| 29 | Protein marker (AMDIS) | 70, 83, **98**, 140, 168 | 18.0 |
| 30 | Protein marker (AMDIS) | 98, 112, 139, 168, **198** | 19.9 |
| 31 | ? | 100, 169, **198**, 205 | 21.9 |
| 32 | ? | 122, 135, 150, 191, **219** | 22.2 |
| 33 | ? | **70**, 100, 124, 194, 224 | 23.3 |
| 34 | Cyclo(Pro-Hyp) | **70**, 86, 124, (210) | 24.6 |
| 35 | ? | 188, **217**, 245 | 26.8 |
| 36 | ? | 196, 210, 224, **239** | 27.1 |
| 37 | ? | **94**, 150 | 16.5* |
| 38 | Cyclo(Pro-Ala)1 | **70**, 97, 125, (168) | 16.7* |
| 39 | Cyclo(Pro-Ala)2 | **70**, 97, 125, (168) | 17.0* |
| 40 | Pro-Arg derivative | 54, 66, 94, **176** | 17.3* |
| 41 | ? | 66, **94**, 185 | 17.9* |
| 42 | ? | 67, 95, 123, **152** | 18.1* |
| 43 | ? | 94, 189, **204** | 18.7* |
| 44 | ? | 90, **117**, 186 | 20.8* |
| 45 | ? | 68, 86, **170** | 22.3* |
| 46 | ? | 89, 94, 117, 237, **238** | 25.2* |
| 47 | Phenol | 66, (**94**) | 8.3 |

| | | | |
|---|---|---|---|
| 48 | Methylphenol | 77, 90, **107**, (108) | 9.7 |
| 49 | Methylindole | 77, 103, **130**, (131) | 14.2 |
| 50 | ? | **91**, 100, 147, 182 | 15.7 |
| 51 | Cyclo(Pro-Val)1 | 70, 72, 125, **154** | 19.7 |
| 52 | Cyclo(Pro-Val)2 | 70, 72, 125, **154** | 20.2 |
| 53 | Cyclo(Pro-Ile)1 | 70, 86, 125, **154** | 20.9 |
| 54 | Cyclo(Pro-Ile)2 | 70, 86, 125, **154** | 21.2 |
| 55 | Cyclo(Pro-Ile)3 | 70, 86, 125, **154** | 21.4 |
| 56 | Cyclo(Pro-pyroGlu) | **70**, 96, 124, 152, 180, 208 | 25.9 |
| 57 | Cyclo(Pro-Phe)1 | 70, 91, 125, **153**, (244) | 26.7 |
| 58 | Cyclo(Pro-Phe)2 | 70, 91, 125, **153**, (244) | 27.1 |
| 59 | Methyl pyrrole-2-carboxylate | 66, **94**, (125) | 10.3 |
| 60 | ? | 79, 108, 120, **139** | 11.8 |
| 61 | 5-oxo-l-proline methyl ester | 56, **84**, (143) | 14.0 |
| 62 | (*E*)-3-Phenylpropenenitrile | 102, (**129**) | 11.4 |
| 63 | 2-Cyano-benzoic acid | 50, 76, 104, (**147**) | 15.4 |
| 64 | Hexadecanenitrile | **43**, 57, 97, 110, 194, 222, (236) | 20.0 |
| 65 | Octadecanitrile | **43**, 57, 97, 110, 194, 222, 236, (265) | 22.6 |
| 66 | Benzoic acid methyl ester | 51, 77, **105**, (136) | 9.9 |
| 67 | 1-Methyl-2,5-pyrrolidinedione | 56, (**113**) | 10.0 |
| 68 | ? | 56, 99, 100, **128** | 11.6 |
| 69 | ? | **55**, 140 | 11.7 |
| 70 | ? | **68**, 69, 138 | 11.8 |
| 71 | ? | 69, **91**, 124, 181, 196 | 12.1 |
| 72 | ? | **58**, 135, 136 | 12.4 |
| 73 | ? | 54, **127**, 128 | 12.7 |
| 74 | ? | 113, **128**, 170 | 13.2 |
| 75 | ? | **68**, 69, 108, 154 | 13.5 |
| 76 | ? | **56**, 125, 137, 152 | 13.8 |
| 77 | ? | 103, **114**, 131, 162 | 14.0 |

| | | | |
|---|---|---|---|
| 78 | 1,3,5-Trimethyl-2,4(1H,3H)-pyrimidinedione derivative | 68, **69**, 126, (154) | 14.3 |
| 79 | ? | 113, 127, **128**, 141 | 14.5 |
| 80 | Methylphthalimide | 76, 104, 117, (**161**) | 14.7 |
| 81 | N-Methyl-5-hydroxyindole (derivative) | **118**, 132, 146, 161 | 14.8 |
| 82 | ? | 125, **153**, 154, 196 | 16.7 |
| 83 | ? | 111, 137, **152**, 196 | 17.2 |
| 84 | 1-Phenyl-1-penten-3-one | 77, **103**, 131, (160) | 18.2 |
| 85 | N-(p-Vinylbenzoyl)-l-alanine | 77, 103, **131**, 175 | 18.3 |
| 86 | Cyclo(Phe-Ala) | 91, **127**, 218 | 19.3 |
| 87 | ? | 89, 116, 131, **216** | 20.8 |
| 88 | ? | 124, 146, 174, 181, **246**, 272 | 24.6 |

*Stankiewicz et al. (1997) report this as Cyclo(Pro-Hyp), however Fabbri et al. (2012) and Orsini et al. (2017) report a different mass spectrum for this DKP (the one corresponding to peak 33). Various papers were consulted for identification[4–11].

**Table S2.** Bulk $\delta^{13}C$ and $\delta^{15}N$ values for reference proteinaceous binders determined by EA-IRMS analysis. The values shown are the mean from repeated measurements ($n$ = 3, except for GRS2 where $n$ = 2 due to an IRMS fault) along with their SD.

| Reference proteinaceous binder | $\delta^{13}C$ (‰) | $\delta^{15}N$ (‰) |
|---|---|---|
| GRS1 | –22.0 (± 0.02) | 5.7 (± 0.2) |
| GRS2 | –21.8 (± 0.2) | 5.2 (± 0.2) |
| GI1 | –10.0 (± 0.1) | 12.0 (± 0.03) |
| GI2 | –11.7 (± 0.8) | 13.4 (± 0.2) |
| C2 | –16.2 (± 0.04) | 5.5 (± 0.1) |
| EWC | –24.5 (± 0.1) | 3.8 (± 0.02) |
| EWD | –25.3 (± 0.1) | 6.3 (± 0.04) |
| EYC | –28.4 (± 0.1) | 5.1 (± 0.1) |
| EYD | –28.4 (± 0.01) | 6.4 (± 0.1) |

**Table S3**. Compound-specific $\delta^{13}C$ values for AAs of animal glues and casein references, determined by GC-C-IRMS analysis. The values shown are the mean from duplicate measurements ($n = 2$), along with their SD. Lys was excluded due to poor chromatography.

| **AA** | **$\delta^{13}C$ (‰) values of reference proteinaceous binder** | | | | |
|---|---|---|---|---|---|
| | **GRS1** | **GRS2** | **GI1** | **GI2** | **C2** |
| **Ala** | –20.8 (± 0.3) | –20.9 (± 0.2) | –12.0 (± 0.3) | –15.6 (± 0.2) | –14.6 (± 0.3) |
| **Gly** | –19.5 (± 0.2) | –19.8 (± 0.2) | –3.7 (± 0.3) | –9.4 (± 0.2) | –10.4 (± 0.2) |
| **Val** | –37.1 (± 0.3) | –36.6 (± 0.3) | –31.5 (± 0.3) | –33.3 (± 0.2) | –29.9 (± 0.3) |
| **Leu** | –32.4 (± 0.2) | –32.8 (± 0.2) | –21.7 (± 0.2) | –24.9 (± 0.5) | –26.2 (± 0.2) |
| **Ile** | –25.7 (± 0.8) | –26.6 (± 0.2) | –16.0 (± 2.7) | –22.7 (± 1.6) | –21.6 (± 0.2) |
| **Nle** | –27.6 (± 0.2) | –27.9 (± 0.4) | –28.2 (± 0.2) | –25.2 (± 0.2) | –25.6 (± 0.2) |
| **Thr** | –13.1 (± 0.4) | –12.8 (± 0.3) | –9.5 (± 0.7) | –10.0 (± 0.3) | –5.6 (± 2.5) |
| **Ser** | –16.6 (± 0.2) | –14.8 (± 0.4) | –0.7 (± 0.1) | –6.2 (± 0.4) | –9.9 (± 0.4) |
| **Pro** | –23.2 (± 0.2) | –23.2 (± 0.2) | –13.6 (± 0.2) | –17.0 (± 0.1) | –17.2 (± 0.1) |
| **Asx** | –20.7 (± 0.1) | –20.3 (± 0.2) | –11.1 (± 0.1) | –16.6 (± 0.2) | –10.7 (± 0.1) |
| **Glx** | –21.8 (± 0.2) | –21.9 (± 0.2) | –11.7 (± 0.1) | –18.5 (± 0.1) | –12.1 (± 0.2) |
| **Hyp** | –22.3 (± 0.1) | –22.5 (± 0.3) | –13.1 (± 0.1) | –16.4 (± 0.2) | NA |
| **Phe** | –29.4 (± 0.1) | –29.6 (± 0.4) | –22.4 (± 0.6) | –27.6 (± 0.4) | –23.5 (± 0.2) |

**Table S4.** Compound-specific δ$^{13}$C values for AAs of chicken and duck egg references, determined by GC-C-IRMS analysis. The values shown are the mean from duplicate measurements (*n* = 2), along with their SD. Lys was excluded due to poor chromatography.

| AA | δ$^{13}$C (‰) values of reference proteinaceous binder | | | |
|---|---|---|---|---|
| | EYC | EYD | EWC | EWD |
| **Ala** | –20.8 (± 0.2) | –2.2 (± 0.5)* | –21.0 (± 0.2) | –22.9 (± 0.2) |
| **Gly** | –19.6 (± 0.3) | –21.0 (± 0.2) | –18.8 (± 0.2) | –21.2 (± 0.2) |
| **Val** | –36.1 (± 0.2) | –36.9 (± 0.2) | –36.3 (± 0.2) | –38.9 (± 0.3) |
| **Leu** | –33.4 (± 0.2) | –34.8 (± 0.2) | –33.3 (± 0.2) | –35.6 (± 0.2) |
| **Ile** | –26.3 (± 0.3) | –27.6 (± 0.2) | –27.0 (± 0.3) | –29.4 (± 0.2) |
| **Nle** | –28.1 (± 0.2) | –28.5 (± 0.4) | –28.0 (± 0.2) | –28.5 (± 0.2) |
| **Thr** | –14.4 (± 0.4) | –17.3 (± 0.3) | –14.4 (± 1.5) | –21.8 (± 1.0) |
| **Ser** | –16.9 (± 0.2) | –17.3 (± 0.1) | –17.5 (± 0.3) | –19.9 (± 0.1) |
| **Pro** | –24.8 (± 0.2) | –25.6 (± 0.3) | –25.1 (± 0.2) | –25.0 (± 0.1) |
| **Asx** | –16.6 (± 0.1) | –19.4 (± 0.7) | –21.0 (± 0.2) | –25.3 (± 0.1) |
| **Glx** | –21.3 (± 0.2) | –22.9 (± 0.2) | –23.3 (± 0.2) | –26.2 (± 0.2) |
| **Hyp** | NA† | NA | NA | NA |
| **Phe** | –28.3 (± 0.1) | –30.1 (± 0.1) | –28.8 (± 0.1) | –31.5 (± 0.2) |

*coeluting with unknown compound, value not reliable; †due to absence of AA-related peak (Hyp)

**Table S5.** Compound-specific δ$^{13}$C values for DKPs of reference proteinaceous binders, determined by DIP-GC-C-IRMS analysis. The value shown is a mean between replicate measurements (for GRS1 *n* = 7, GRS2 and GI1 *n* = 2, GI2 *n* = 6) along with their SD.

| Animal glue | Theoretical δ$^{13}C_{DKPT}$ values (‰) | | | Experimental δ$^{13}C_{DKP}$ values (‰) | | |
|---|---|---|---|---|---|---|
| | Pyr-Pyr | Pro-Gly | Pro-Hyp | Pyr-Pyr | Pro-Gly | Pro-Hyp |
| **GRS1** | –22.3 (± 0.1) | –22.1 (± 0.2) | –22.8 (± 0.1) | –21.7 (± 0.6) | –19.0 (± 0.8) | –19.8 (± 0.9) |
| **GRS2** | –22.5 (± 0.2) | –22.2 (± 0.2) | –22.9 (± 0.2) | –21.8 (± 0.9) | –18.9 (± 0.4) | –20.9 (± 0.9) |
| **GI1** | –13.1 (± 0.1) | –10.8 (± 0.2) | –13.4 (± 0.1) | –11.8 (± 0.2) | –6.1 (± 0.5) | –9.7 (± 0.2) |
| **GI2** | –16.4 (± 0.1) | –14.8 (± 0.1) | –16.7 (± 0.1) | –11.7 (± 1.6) | –9.7 (± 0.8) | –13.3 (± 0.4) |

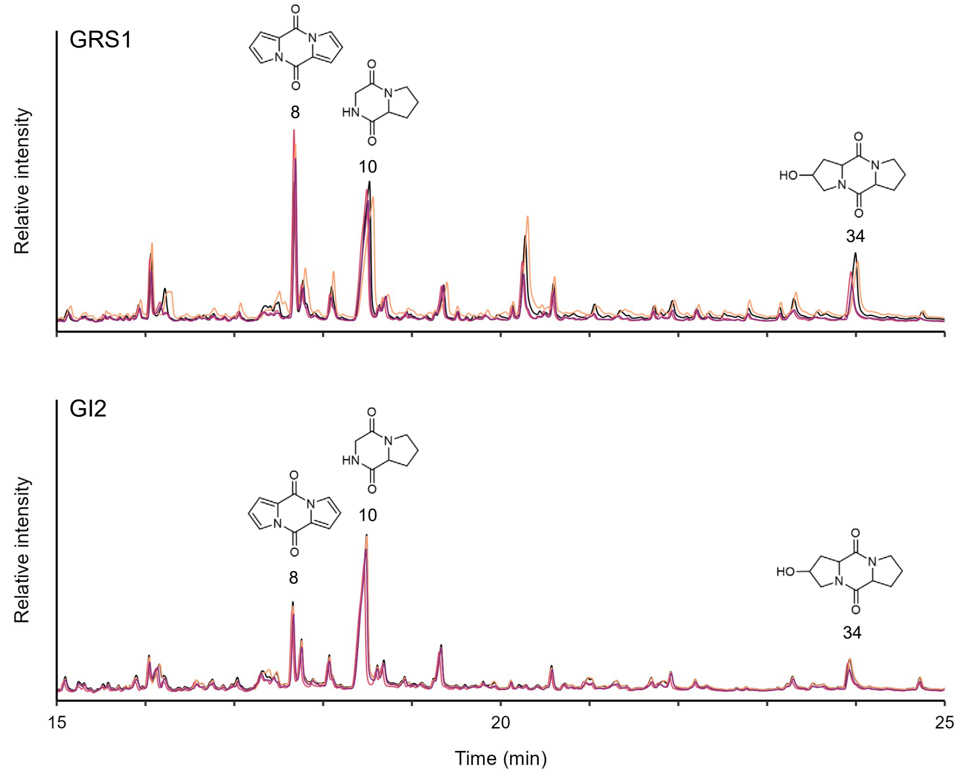


**Figure S4.** Partial DIP-GC-C-IRMS chromatograms, displaying the ion current signal for *m/z* 44 in black, purple, pink, and orange, obtained from four repeated analyses of rabbit skin glue, GRS1, and four repeated analyses of isinglass glue, GI2. For each glue, signals are shown at a common instrument-generated intensity scale. Relevant DKPs, for which isotopic determinations were carried out, are indicated with their structures; 8 = cyclo(Pyr-Pyr), 10 = cyclo(Pro-Gly), 34 = cyclo(Pro-Hyp).

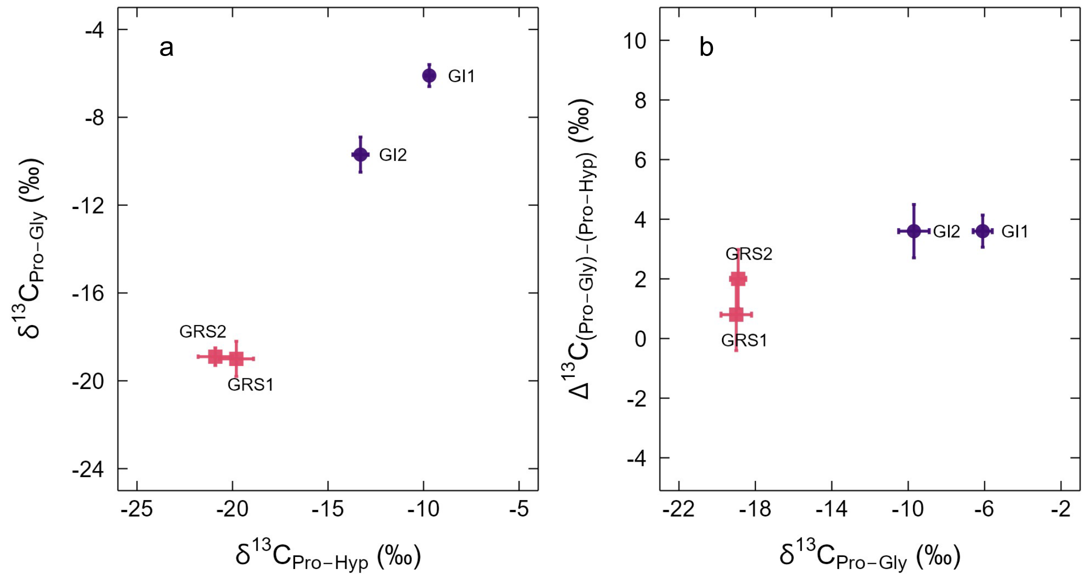


**Figure S5.** (a) Plot of the $\delta^{13}C_{Pro\text{-}Gly}$ values for isinglass glues (GI1 and GI2, blue circles) and rabbit skin glues (GRS1 and GRS2, pink squares), plotted against their $\delta^{13}C_{Pro\text{-}Hyp}$ values. (b) Plot showing the $\Delta^{13}C_{(Pro\text{-}Gly)-(Pro\text{-}Hyp)}$ values, for the same glues, plotted against their $\delta^{13}C_{Pro\text{-}Gly}$ values. The error bars represent the SD on either side (±) of the value. Instrumental precision was 0.3‰.

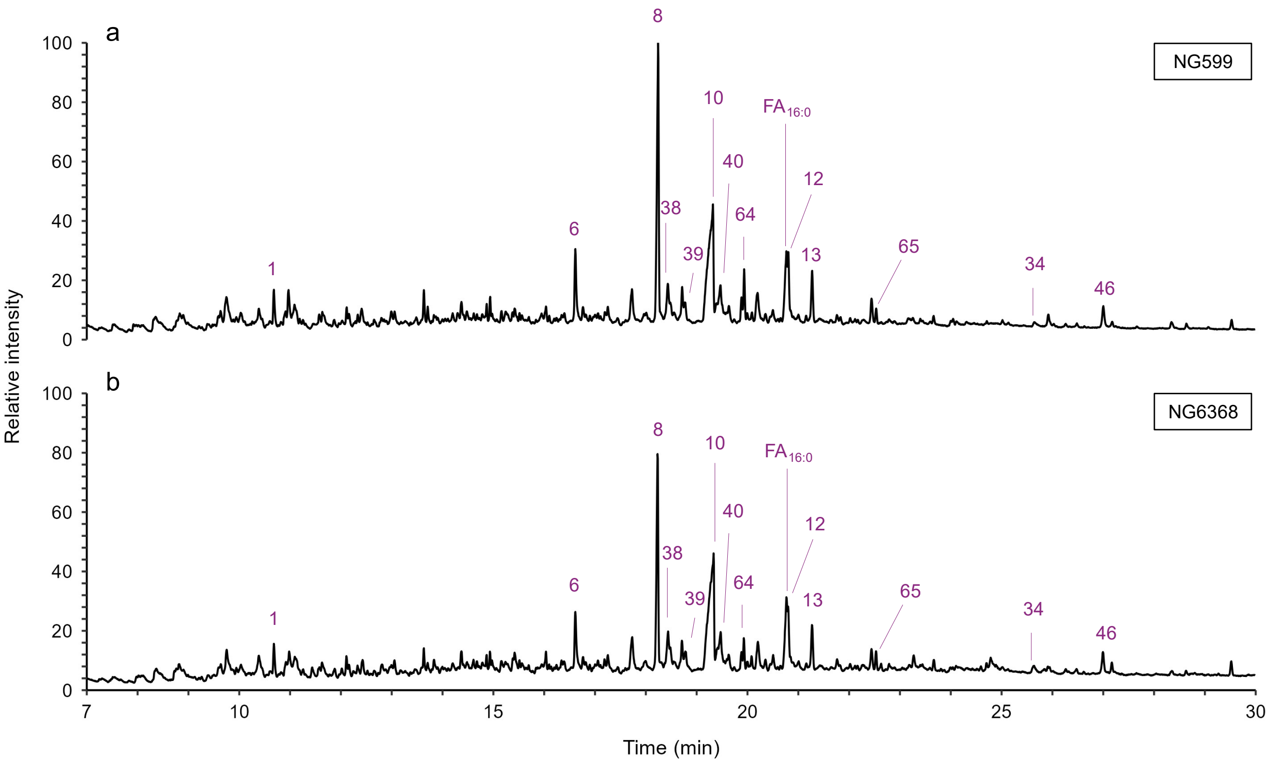


**Figure S6**. Partial DIP-GC-MS TIC of the ground layer from NG599 (a) and NG6368 (b). Relevant information for the corresponding peak numbers is listed in Table S1.

## References


(1) Geddes da Filicaia, E. Innovative Stable Isotope and High-Resolution Mass Spectrometry Approaches for Provenancing Organic Materials in Paintings and Archaeological Artefacts, University of Bristol, 2025.

(2) Coplen, T. B.; Böhlke, J. K.; Bièvre, P. D.; Ding, T.; Holden, N. E.; Hopple, J. A.; Krouse, H. R.; Lamberty, A.; Peiser, H. S.; Revesz, K.; Rieder, S. E.; Rosman, K. J. R.; Roth, E.; Taylor, P. D. P.; Vocke, R. D.; Xiao, Y. K. Isotope-Abundance Variations of Selected Elements (IUPAC Technical Report). *Pure and Applied Chemistry* **2002**, *74* (10), 1987–2017. https://doi.org/10.1351/pac200274101987.

(3) Corr, L. T.; Berstan, R.; Evershed, R. P. Optimisation of Derivatisation Procedures for the Determination of $\delta^{13}C$ Values of Amino Acids by Gas Chromatography/Combustion/Isotope Ratio Mass Spectrometry. *Rapid Commun. Mass Spectrom.* **2007**, *21* (23), 3759–3771. https://doi.org/10.1002/rcm.3252.

(4) Stankiewicz, B. A.; Hutchins, J. C.; Thomson, R.; Briggs, D. E. G.; Evershed, P. Assessment of Bog-Body Tissue Preservation by Pyrolysis-Gas Chromatography/Mass Spectrometry. *Rapid Communications in Mass Spectrometry* **1997**, *11*, 7.

(5) Fabbri, D.; Adamiano, A.; Falini, G.; De Marco, R.; Mancini, I. Analytical Pyrolysis of Dipeptides Containing Proline and Amino Acids with Polar Side Chains. Novel 2,5-Diketopiperazine Markers in the Pyrolysates of Proteins. *Journal of Analytical and Applied Pyrolysis* **2012**, *95*, 145–155. https://doi.org/10.1016/j.jaap.2012.02.001.

(6) Adamiano, A.; Fabbri, D.; Falini, G.; Giovanna Belcastro, M. A Complementary Approach Using Analytical Pyrolysis to Evaluate Collagen Degradation and Mineral Fossilisation in Archaeological Bones: The Case Study of Vicenne-Campochiaro Necropolis (Italy). *Journal of Analytical and Applied Pyrolysis* **2013**, *100*, 173–180. https://doi.org/10.1016/j.jaap.2012.12.014.

(7) Orsini, S.; Parlanti, F.; Bonaduce, I. Analytical Pyrolysis of Proteins in Samples from Artistic and Archaeological Objects. *Journal of Analytical and Applied Pyrolysis* **2017**, *124*, 643–657. https://doi.org/10.1016/j.jaap.2016.12.017.

(8) Smith, G. G.; Reddy, G. S.; Boon, J. J. Gas Chromatographic–Mass Spectrometric Analysis of the Curie-Point Pyrolysis Products of Some Dipeptides and Their Diketopiperazine. *J. Chem. Soc., Perkin Trans. 2* **1988**, No. 2, 203–211. https://doi.org/10.1039/P29880000203.

(9) Choi, S.-S.; Ko, J.-E. Dimerization Reactions of Amino Acids by Pyrolysis. *Journal of Analytical and Applied Pyrolysis* **2010**, *89* (1), 74–86. https://doi.org/10.1016/j.jaap.2010.05.009.

(10) Choi, S.-S.; Ko, J.-E. Analysis of Cyclic Pyrolysis Products Formed from Amino Acid Monomer. *Journal of Chromatography A* **2011**, *1218* (46), 8443–8455. https://doi.org/10.1016/j.chroma.2011.09.055.

(11) Gallois, N.; Templier, J.; Derenne, S. Pyrolysis-Gas Chromatography–Mass Spectrometry of the 20 Protein Amino Acids in the Presence of TMAH. *Journal of Analytical and Applied Pyrolysis* **2007**, *80* (1), 216–230. https://doi.org/10.1016/j.jaap.2007.02.010.
